# Preemptive optimization of a clinical antibody for broad neutralization of SARS-CoV-2 variants and robustness against viral escape

**DOI:** 10.1126/sciadv.adu0718

**Published:** 2025-03-28

**Authors:** Fangqiang Zhu, Saravanan Rajan, Conor F. Hayes, Ka Yin Kwong, Andre R. Goncalves, Adam T. Zemla, Edmond Y. Lau, Yi Zhang, Yingyun Cai, John W. Goforth, Mikel Landajuela, Pavlo Gilchuk, Michael Kierny, Andrew Dippel, Bismark Amofah, Gilad Kaplan, Vanessa Cadevilla Peano, Christopher Morehouse, Ben Sparklin, Vancheswaran Gopalakrishnan, Kevin M. Tuffy, Amy Nguyen, Jagadish Beloor, Gustavo Kijak, Chang Liu, Aiste Dijokaite-Guraliuc, Juthathip Mongkolsapaya, Gavin R. Screaton, Brenden K. Petersen, Thomas A. Desautels, Drew Bennett, Simone Conti, Brent W. Segelke, Kathryn T. Arrildt, Samantha Kaul, Emilia A. Grzesiak, Felipe Leno da Silva, Thomas W. Bates, Christopher G. Earnhart, Svetlana Hopkins, Shivshankar Sundaram, Mark T. Esser, Joseph R. Francica, Daniel M. Faissol, LLNL Generative Unconstrained Intelligent Drug Engineering (GUIDE) consortium

**Affiliations:** ^1^ Biosciences and Biotechnology Division, Physical and Life Sciences Directorate, Lawrence Livermore National Laboratory, Livermore, CA 94550, USA.; ^2^ Biologics Engineering, R&D, AstraZeneca, Gaithersburg, MD 20878, USA.; ^3^ Computational Engineering Division, Lawrence Livermore National Laboratory, Livermore, CA 94550, USA.; ^4^ Global Security Computing Applications Division, Lawrence Livermore National Laboratory, Livermore, CA 94550, USA.; ^5^ Vaccines and Immune Therapies, BioPharmaceuticals R&D, AstraZeneca, Gaithersburg, MD 20878, USA.; ^6^ Chinese Academy of Medical Science (CAMS) Oxford Institute, University of Oxford, Oxford OX3 7BN, UK.; ^7^ Centre for Human Genetics, Nuffield Department of Medicine, University of Oxford, Oxford OX3 7BN, UK.; ^8^ Mahidol-Oxford Tropical Medicine Research Unit, Bangkok, Thailand.; ^9^ Joint Program Executive Office for Chemical, Biological, Radiological, and Nuclear Defense, US Department of Defense, Frederick, MD 21703, USA.; ^10^ Joint Research and Development Inc., Stafford, VA 22556, USA.; ^11^ Center for Bioengineering, Lawrence Livermore National Laboratory, Livermore, CA 94550, USA.

## Abstract

Most previously authorized clinical antibodies against severe acute respiratory syndrome coronavirus 2 (SARS-CoV-2) have lost neutralizing activity to recent variants due to rapid viral evolution. To mitigate such escape, we preemptively enhance AZD3152, an antibody authorized for prophylaxis in immunocompromised individuals. Using deep mutational scanning (DMS) on the SARS-CoV-2 antigen, we identify AZD3152 vulnerabilities at antigen positions F456 and D420. Through two iterations of computational antibody design that integrates structure-based modeling, machine-learning, and experimental validation, we co-optimize AZD3152 against 24 contemporary and previous SARS-CoV-2 variants, as well as 20 potential future escape variants. Our top candidate, 3152-1142, restores full potency (100-fold improvement) against the more recently emerged XBB.1.5+F456L variant that escaped AZD3152, maintains potency against previous variants of concern, and shows no additional vulnerability as assessed by DMS. This preemptive mitigation demonstrates a generalizable approach for optimizing existing antibodies against potential future viral escape.

## Teaser

Computational optimization based on anticipated vulnerability substantially enhanced antibody AZD3152 against SARS-CoV-2 variants.

## INTRODUCTION

Despite available vaccines (*1*), COVID-19 remains a severe health threat (*2*) for individuals who have an impaired immune response to vaccination, thus necessitating continued vigilance for discovery of alternative medicines. Initially, monoclonal antibody (mAb) therapies offered a promising avenue for severe acute respiratory syndrome coronavirus 2 (SARS-CoV-2) prophylaxis and treatment (*3*). Rapid viral evolution, however, led to emergence of escape mutations that evade antibody-mediated neutralization (*3*, *4*). Hence, most clinically approved SARS-CoV-2 antibody therapies have lost activity (*5*, *6*). Tixagevimab/cilgavimab (*7*), which was authorized as the first preexposure prophylactic SARS-CoV-2 antibody therapy for the immunocompromised, was withdrawn from use after approximately 1 year because of loss of neutralizing activity against more recent Omicron variants (*8*).

Rapid antigenic drift of SARS-CoV-2 poses a major challenge to vaccine and mAb development, requiring extensive resources to keep pace with emerging variants (*9*). Immunobridging (*10*) strategies have opened a pathway for licensed vaccines, such as those for seasonal influenza, to be updated and reauthorized if results from an immunological bridging study demonstrate that the updated vaccine has correlates of protection similar to the originally licensed vaccine. With a shortened clinical testing cycle, derivative vaccines can rapidly address emerging variants of concern (VOCs). It is conceivable that a similar approach could be applied to authorization of derivative clinical antibodies, particularly if the derivative and licensed antibodies differ by only a few amino acids. As recently demonstrated by the computational redesign of the antibody COV2-2130 (parental mAb of cilgavimab) (*11*), where computational tools were used to identify four amino acid substitutions that successfully restored efficacy against Omicron escape variants, antibody therapeutics can be modified to mitigate escape due to antigenic drift. In addition, deep mutational scanning (DMS) (*12*–*14*) enables the identification of mAb vulnerabilities such that antigen escape mutations can be anticipated. Combining computational antibody optimization with information gathered from DMS could facilitate the rapid discovery of derivative mAbs that guard against viral escape.

AZD3152 (*15*), an antibody targeting SARS-CoV-2, has been recently developed to provide preexposure prophylaxis for immunocompromised individuals. AZD3152 neutralizes SARS-CoV-2 by binding to the receptor binding domain (RBD) of spike protein, as revealed by a recent x-ray crystal structure [Protein Data Bank (PDB) code: 8SUO; 3.3-Å resolution] of the AZD3152 antigen-binding fragment (Fab) in complex with the Omicron BA.2 RBD (*15*). However, the continuing emergence of SARS-CoV-2 variants necessitates a proactive approach for guarding against viral escape. Here, we report the successful preemptive optimization of AZD3152 aimed at increasing robustness to future escape mutations. This was achieved by integrating results from DMS with computational design and in vitro validation (fig. S2A). DMS was used to identify potential escape mutations; structure-guided computational design was used to propose antibody derivatives predicted to bind to escape variants with improved affinity; in vitro assays were used to validate and screen computationally designed mAbs.

## RESULTS

### DMS revealed potential vulnerabilities of AZD3152

**Fig. 1.**
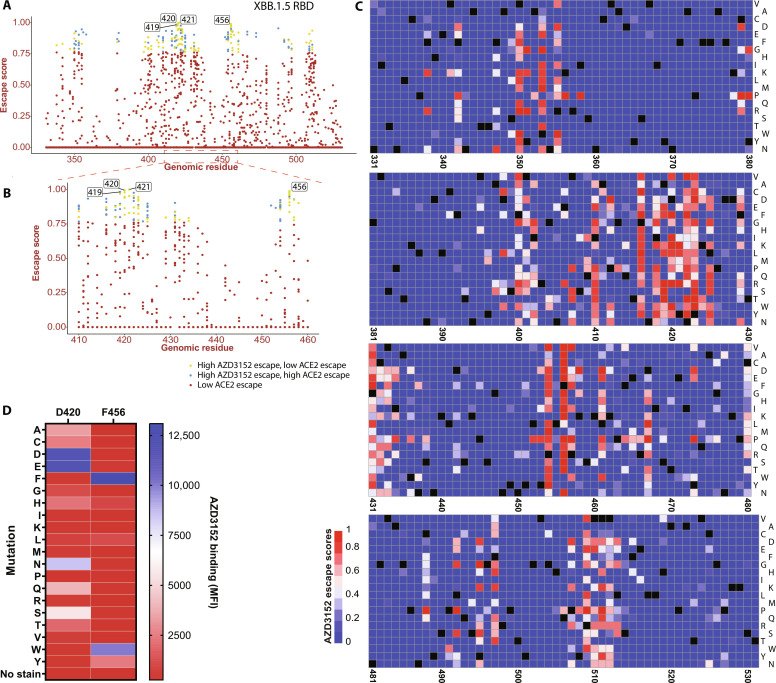
DMS analysis of AZD3152. (**A** and **B**) Distribution of escape scores per residue of the SARS-CoV-2 XBB.1.5 RBD. High and low escape from AZD3152 and ACE2 were classified at the 95th percentile of the distribution. (**C**) Heatmap illustrating the impact of every mutation on AZD3152 binding. Black squares represent the wild-type amino acid for a particular residue. (**D**) Validation of DMS results. Yeast displaying all mutations at position 420 and 456 (XBB.1.5) were profiled for binding to AZD3152 by flow cytometry. MFI, median fluorescence intensity.

Using a library of antigen variants with single mutations to XBB.1.5 RBD, DMS was applied to determine potential escape vulnerabilities of AZD3152 (section S1.1). Briefly, each of the 201 amino acids of the XBB.1.5 RBD was randomized to all 20 amino acids by replacing each codon with the NNK codon (data file S1), and the cloned library (4 × 10^3^ members) was displayed on the surface of *Saccharomyces cerevisiae*. The library was subjected to next-generation sequencing (NGS), confirming that all expected amino acid mutations for each position were present. Through yeast sorting, we collected cells that stained positive for RBD expression relative to an unstained control but showed no binding to AZD3152. We then applied NGS to obtain the RBD sequences in such cells, thereby identifying potential escape mutations (fig. S2B). Escape scores were calculated for each mutation of each RBD residue as the differential read count of RBD mutants in the presort versus post-sort conditions. Any mutation with a score above the 95th percentile of the distribution was classified as having a “high escape potential.” Accordingly, we identified the residues A419, D420, Y421, and F456 as having mutations with the greatest escape score (Fig. 1), consistent with class 1 epitope for SARS-CoV-2 RBD (*13*, *14*). As an additional down-selection step, we further investigated the impact of these mutations to escape ACE2 binding and found that the virus could maintain ACE2 binding when incorporating mutations at the aforementioned four residues. Last, we characterized the overall impact of these residues on mAb and ACE2 binding by summing the escape scores for all mutations at a residue (fig. S3) and noticed that positions F456 and D420 had the highest overall escape scores for AZD3152 binding but low escape scores for ACE2.

DMS results were further validated by constructing all possible single-point mutants at D420 and F456 on XBB.1.5 RBD and testing the mutant RBDs for AZD3152 binding (section S1.2). We confirmed that mutants at these positions that have high DMS escape scores also lose binding in the monoclonal yeast-binding assay. In particular, mutating F456 to anything other than an aromatic amino acid results in total loss of binding to AZD3152 (Fig. 1D).

### First design iteration evaluated diverse sets of antibody mutations and antigens

To address AZD3152 vulnerabilities to D420 and F456 mutations, we used the Generative Unconstrained Intelligent Drug Engineering (GUIDE) computational engine [previously described (*11*)] with some additional enhancements described below. For our first design iteration, we sought to identify AZD3152 derivatives with increased breadth, binding to the maximum number of potential future escape variants without compromising efficacy to recent and previous variants that widely circulated. We used two Omicron variants, BA.2 and XBB, as background antigens for our calculations. On each background, we introduced a number of single-point mutations (section S2.2.3) consisting of those present in contemporary or previous variants (table S2) and five escape mutations at positions D420 and F456 each, as identified by the DMS results. The wild-type background antigens along with the single-point mutants comprise a total of 44 antigens (section S2.2.3) considered in this design iteration.

Using GUIDE, we applied a set of structure-based tools (section S2.1), as recently reported (*11*), to estimate the change in binding free energy, ∆∆*G*, for AZD3152 derivatives. Briefly, free-energy perturbation (FEP) (*16*) and potential of mean force (PMF) (*17*) methods, by using compute-intensive molecular dynamics (MD) simulations, calculate ∆∆*G* from first principles. Rosetta Flex (*18*) offers empirical ∆∆*G* estimation with much lower computational cost compared to FEP or PMF, thus making large quantities of binding predictions more tractable. The structural fluctuation estimation (SFE) method (*19*) is aimed at improving the robustness of ∆∆*G* calculations to structural uncertainties and fluctuations by sampling many conformations from equilibrium MD trajectories initiated with different structural models and then calculating ∆∆*G* via Rosetta Flex after energy minimization. Approximately 137,000 AZD3152-derived sequences were evaluated by one or more of these structure-based ∆∆*G* calculation methods (further details in section S2.1). In addition to predicting changes in binding affinity, we also evaluated the effects of single mutations on conformational stability of the antibody using FEP (*16*). In total, accounting for the 44 antigen targets along with multiple structural models and ∆∆*G* prediction methods, for any given antibody derivative, we computed up to 188 different ∆∆*G* values as described in table S6.

To tackle the complexity arising from the many targets, we treated antibody design as a multiobjective optimization problem to identify candidates from an enormous search space, driven by the predictions from FEP, SFE, PMF, and Rosetta Flex described above. We adopted algorithms based on empirical Pareto front and randomized integer linear programming to generate a large pool of antibody derivatives (detailed in section S2.2), which were subsequently down-selected using utility functions (tables S6 and S7) described below.

Given that it may be biophysically impossible to attain full potency to all prior, contemporary, and future SARS-CoV-2 variants in a single antibody template, our goal was to maximize variant coverage. Further, we did not have knowledge of which, if any, escape mutations might emerge in the future, thus introducing a challenge of not having a clear set of optimization targets. To address this, we developed utility functions that specifically allowed for the optimization of subsets of escape variants without defining those subsets a priori. That is, while all 44 antigen variants were included in the optimization, the choice of which targets to optimize in a given subset was an output of the optimization approach, driven by the ∆∆*G* predictions across all of the variants. The final batch of proposed antibodies represents multiple goals with increasing degrees of breadth. Given that our affinity predictions are not always accurate, we also applied various parameterized constraints (section S2.2.5.1.2.1) throughout the procedure to ensure that the selected batch of candidates has broad sequence diversity.

Using the in silico approaches described above, we selected a set of 187 candidates from a search space of ~10^10^ computationally generated antibody derivatives (section S2.2.2). An additional objective of this design iteration was to produce experimental data to inform a potential second design iteration. In the selection of the 187 candidates, we therefore included many derivatives with small numbers of amino acid substitutions to the parental AZD3152.

**Fig. 2.**
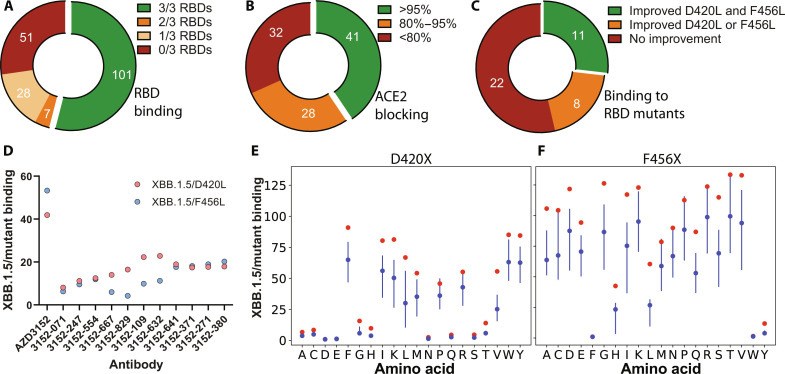
Characterization of candidates from the first design iteration. AZD3152 derivatives underwent a screening cascade to select for candidates (**A**) binding to three SARS-CoV-2 RBD variants (Wuhan, BQ.1.1, and XBB.1.5), (**B**) blocking ACE2-XBB.1 RBD interactions at a level similar to parental AZD3152, and (**C**) showing improved binding to both XBB.1.5 sentinel mutants D420L and F456L. Pie slices indicate number of sequences in each of the annotated categories. (**D**) Fluorescence-activated cell sorting (FACS)–based binding fold changes for 11 derivatives to yeast displaying XBB.1.5+D420L or XBB.1.5+F456L compared to XBB.1.5. (**E** and **F**) Fold changes for the binding of AZD3152 and derivatives to the full panel of single-point mutants at RBD positions D420 (E) and F456 (F). For each RBD mutation, a red dot represents reduction in binding for the parental AZD3152, and a blue vertical line with a dot indicates the range and the median value for the derivatives, respectively.

The 187 selected antibody sequences were subcloned into immunoglobulin G (IgG) expression vectors and transiently expressed in human embryonic kidney (HEK) 293 cells (section S1.3). On the basis of enzyme-linked immunosorbent assay (ELISA) binding (section S1.4) to three SARS-CoV-2 RBDs (Wuhan, BQ.1.1, and XBB.1.5), we found that 101 mAbs retained cross-reactivity (Fig. 2A) and discarded the remainder. Potency of the 101 mAbs was assessed using a high-throughput flow-based ACE2 blocking assay by first saturating RBD-coupled beads with antibody and then measuring binding to fluorescently labeled hACE2 (section S1.5). Forty-one antibodies showed greater than 95% ACE2 blocking activity and were retained for further testing (Fig. 2B).

Subsequently, as our goal was to mitigate escape vulnerabilities to mutants at positions 420 and 456, we performed an initial screen focusing on two sentinel mutations, D420L and F456L on XBB.1.5. Our DMS and confirmatory flow cytometry indicated weak binding of the XBB.1.5+D420L and XBB.1.5+F456L antigens to the parental AZD3152 (Fig. 1), thus making it feasible to detect improved binding among our selected AZD3152 derivatives. We designed a high-throughput assay to assess the relative binding of each mAb to XBB.1.5 versus the sentinel mutation D420L or F456L in a single well to control for well-to-well antibody expression differences (section S1.2). A constitutively expressing enhanced green fluorescent protein (eGFP) construct was transduced into yeast displaying XBB.1.5 and cocultured with an equivalent amount of nonfluorescent yeast displaying either D420L or F456L. By comparing geometric means of GFP-positive (XBB.1.5) and GFP-negative (sentinel mutants) populations, we measured the differences in relative binding for all our antibody candidates. Qualitatively consistent with our previous DMS data (Fig. 1), the parental AZD3152 displayed 42-fold and 53-fold reduced binding to D420L and F456L, respectively, compared to XBB.1.5. Of the 41 candidates selected earlier, 18 and 12 mAbs brought the binding reduction to within 25-fold for D420L and F456L (fig. S4), respectively. We selected 11 mAbs to progress that showed improved binding over AZD3152 to both RBD mutants (Fig. 2, C and D).

Next, we profiled binding of the down-selected mAbs to the full panel of mutations at D420 and F456, using the same yeast coculture binding assay. As shown in Fig. 2 (E and F), our designed mAbs improved binding to nearly all the RBD mutations at these two positions that severely compromised AZD3152 binding. Such partial recovery, while encouraging, would still be insufficient for therapeutic efficacy, thus motivating a second iteration of optimization.

### Second design iteration generated AZD3152 derivatives with substantially improved efficacy

Concurrent with the start of our second design iteration, VOCs with escape mutation F456L emerged (*20*), thus compelling an emphasis on rescuing efficacy against F456L VOCs while still optimizing for future escape mutations and maintaining potency to contemporary and previous VOCs. As mentioned earlier, our first design iteration identified many derivatives that improved binding to a wide range of mutations at positions F456 (including F456L) and D420, achieving high breadth but insufficient potency. For the second iteration, a “select, combine, and diversify” approach (section S2.3) was applied as briefly described below. First, on the basis of the experimental results from the first iteration, the top performing mutations were combined into multipoint derivatives using a tiered approach. Then, a genetic algorithm was used to introduce additional derivatives to the batch, resulting in a more diverse set of antibody sequences. Following this process (section S2.3), a set of 188 candidates were chosen for the second design iteration, most of which differ from the parental AZD3152 by four or more amino acids (see fig. S1).

**Fig. 3.**
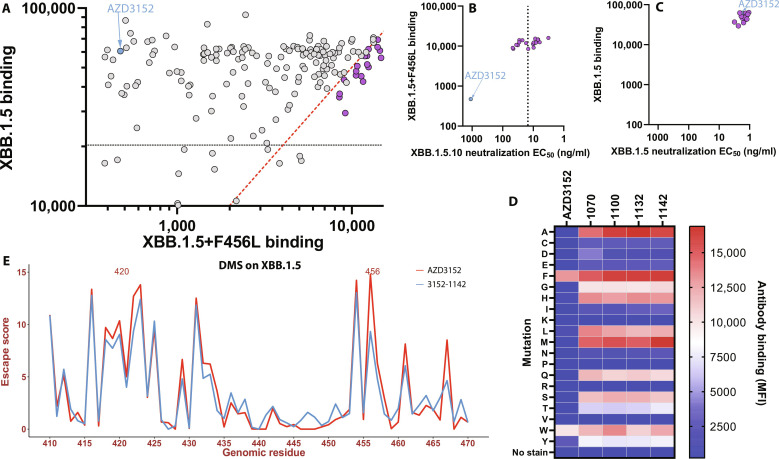
Characterization of candidates from the second design iteration. (**A**) FACS-based binding for AZD3152 mutants on yeast displaying XBB.1.5 and XBB.1.5+F456L identifies 20 lead derivatives. Thresholds for binding are indicated as dashed lines: within threefold from AZD3152 to XBB.1.5 (black) and within fivefold binding of XBB.1.5+F456L over XBB.1.5 (red). (**B** and **C**) Comparison of relative binding and pseudo-neutralization EC_50_ values for the top 20 candidates (purple) compared with parental AZD3152 (blue) for SARS-CoV-2 XBB.1.5.10 (with F456L, B) and XBB.1.5 (without F456L, C). (**D**) Binding of AZD3152 and top four most potent lead derivatives to yeast-displaying XBB.1.5 mutated to all amino acids at position F456 by flow cytometry. (**E**) Comparison of escape scores per RBD residue for AZD3152 (red) and 3152-1142 (blue).

We produced the 188 selected derivatives and tested their differential binding to XBB.1.5+F456L relative to XBB.1.5 using the previously described yeast coculture assay (fig. S5). The screen identified 20 derivatives (Table 1) that bind F456L-displaying yeast within fivefold of XBB.1.5 while maintaining binding to XBB.1.5 within threefold of parental AZD3152 (Fig. 3A). This was a clear improvement from the first iteration, demonstrating the additive benefit of combining favorable amino acid substitutions. Most derivatives contain four to five amino acid substitutions, often at heavy chain (HC) positions 57 and 100 as well as light chain (LC) positions 31, 33, 55, and 95 (Table 1). Crucially, these derivatives rescued binding and potency for XBB.1.5+F456L (Fig. 3B) while retaining binding and potency for XBB.1.5 (Fig. 3C).

**Table 1.** Specific amino acid substitutions in the top 20 derivatives from the second design iteration.

AZD3152 derivatives	Amino acid substitutions
3152-1137	SH57E, AH100E, GL31H, NL33W, GL95H
3152-1135	SH57E, AH100E, NL33H, KL55Q, GL95H
3152-1130	SH57E, AH100E, NL33D, KL55D, GL95H
3152-1131	SH57E, AH100E, NL33H, KL55D, GL95H
3152-1100	SH57E, AH100E, NL33D, GL95H
3152-1142	SH57E, AH100E, NL33D, KL55Q, GL95H
3152-1144	SH57E, AH100E, NL33W, KL55Q, GL95H
3152-1070	SH57E, NL33W, GL95H
3152-1109	SH57E, AH100E, NL33W, GL95H
3152-1132	SH57E, AH100E, NL33D, KL55H, GL95H
3152-1095	AH100R, NL33W, KL55D, GL95H
3152-1138	SH57E, AH100E, GL31W, NL33W, GL95H
3152-1078	SH57E, AH100E, GL31H, NL33W
3152-1120	SH57E, AH100E, NL33H, GL95H
3152-1140	SH57E, AH100E, NL33D, KL55G, GL95H
3152-1105	SH57E, AH100E, NL33Y, GL95H
3152-1107	SH57E, AH100E, KL55D, GL95H
3152-1093	SH57E, AH100E, NL33W, KL55D
3152-1141	SH57E, AH100E, NL33H, KL55G, GL95H
3152-1136	SH57E, AH100E, NL33H, KL55H, GL95H

### 3152-1142 is a potent, broadly neutralizing, and developable antibody

The top 20 derivative antibodies were tested for pseudovirus neutralization (section S1.7) of XBB.1.5 and XBB.1.5.10 (which contains F456L). Compared to the parental AZD3152 [XBB.1.5.10 half maximal effective concentration (EC_50_), 977.2 ng/ml], all 20 derivative mAbs were markedly more potent against XBB.1.5.10 (EC_50_ range of 3.2 to 46.7 ng/ml) while retaining potency against XBB.1.5 (Fig. 3, B and C). Eleven derivative mAbs had XBB.1.5.10 EC_50_ values below 15 ng/ml (Fig. 3B); these were then characterized for developability in a panel of biophysical assays (Table 2). Following protein A purification, we observed that all derivatives had high monomer content by size exclusion chromatography (<95%), with retention times consistent with our NIP228 IgG1 control. All mAbs showed minimal nonspecific binding in a baculovirus particle ELISA (section S1.10) and showed no binding to untransfected HEK293 cells. None of the antibodies exhibited a large red shift in the affinity-capture self-interaction nanoparticle spectroscopy assay in either phosphate-buffered saline or His-Arg buffer, indicating a low risk for reversible self-association. Last, we tested for changes in monomer content following heat or photo stress by incubation at 45°C for 14 days or exposure to 3000 lux cool white light for 7 days. Only 3152-1137 exhibited greater than 5% loss in monomer content following photo stress. However, 7 of 11 mAbs showed large reductions in monomer content following heat stress (Table 2), including two (3152-1131 and 3152-1144) that completely aggregated and formed insoluble precipitates. These seven derivatives were therefore excluded from further consideration.

**Table 2.** Developability profiling of AZD3152 derivatives. ΔRT, change in column retention time versus NIP228 control mAb; BVP, baculovirus particle binding; AC-SINS, affinity-capture self-interaction nanoparticle spectroscopy, measured in PBS (phosphate buffered saline buffer) and HA (histidine-arginine buffer). See sections S1.9 to S1.14 for details.

mAb derivative	% Monomer content after protein A (>95%)	ΔRT from NIP228 (<0.2 m)	BVP score (<5)	HEK293 cell binding	AC-SINS (PBS)	AC-SINS (HA)	14d heat stress change in monomer %	7d photo stress change in monomer %
AZD3152-parental	97.6	0.03	1.16	N	0.5	0.5	1.23	−0.33
3152-1070	98.6	0.10	3.48	N	2	1	−1.05	−1.05
3152-1100	99.3	−0.12	1.46	N	1	0	−1.20	−0.29
3152-1109	99.2	0.05	1.51	N	0	0	−4.55	−0.63
3152-1130	98.8	−0.19	2.23	N	1	0	−46.81	−0.22
3152-1131	97.8	−0.13	3.14	N	0	0	−99.71	−0.31
3152-1132	99.3	−0.11	1.21	N	0	0	−1.04	−0.53
3152-1135	99.3	−0.08	1.29	N	1	4	−5.61	−0.50
3152-1137	96.8	0.17	4.23	N	1	0	−30.78	−5.08
3152-1140	97.8	−0.14	1.61	N	2	1	−18.45	0.00
3152-1142	99.3	−0.14	1.35	N	1	0	−1.15	−0.41
3152-1144	98.5	0.03	2.22	N	2	0	−100	0.00

We profiled the four remaining derivatives (3152-1070, 3152-1100, 3152-1132, and 3152-1142) for binding against the full panel of mutations at F456 to all the 20 amino acids (Fig. 3D). These derivatives were confirmed to bind to 17 of 20 amino acid substitutions at position 456, compared with just 3 of 20 for the parental AZD3152 (Fig. 3D). To select our final lead, we measured pseudovirus neutralization against a large panel of 18 variants through EG.5.1 (Table 3), as well as authentic virus neutralization against a panel of variants through XBB.1.5 (Table 4). Given its breadth, potency (EC_50_ range of 0.5 to 16.6 ng/ml), and developability, 3152-1142 was selected as the lead derivative mAb.

**Table 3.** SARS-CoV-2 antiviral activity of AZD3152 derivatives in pseudovirus neutralization assays. EC_50_ values are calculated from replicates run in at least triplicate. n.t., not tested.

SARS-CoV-2 variants	SARS-CoV-2 pseudovirus neutralization EC_50_ (ng/ml)				
	AZD3152	3152-1070	3152-1100	3152-1132	3152-1142
D614G	14.8 ± 2.8	17.4 ± 5.0	16.8 ± 5.2	15.6 ± 5.2	16.4 ± 4.0
Alpha	9.1 ± 2.5	10.9 ± 4.2	15.4 ± 5.1	10.5 ± 3.6	7.8 ± 2.3
Beta	15.9 ± 5.3	11.1 ± 5.1	16.9 ± 5.7	14.8 ± 4.1	10.9 ± 4.5
Delta	36.6 ± 11.4	24.9 ± 7.6	20.6 ± 5.9	16.5 ± 7.9	16.6 ± 4.4
Gamma	5.1 ± 0.6	4.5 ± 0.5	3.5 ± 0.7	4.4 ± 0.7	3.4 ± 0.5
BA.1	3.5 ± 0.9	1.7 ± 0.6	1.1 ± 0.3	0.8 ± 0.2	0.9 ± 0.3
BA.1.1	3.5 ± 0.8	1.8 ± 0.2	1.9 ± 0.4	1.0 ± 0.3	0.9 ± 0.1
BA.2	9.9 ± 2.2	6.9 ± 1.7	3.5 ± 0.6	1.6 ± 0.3	1.5 ± 0.2
BA.2.12.1	5.6 ± 1.7	3 ± 0.6	1.1 ± 0.3	0.5 ± 0.1	0.5 ± 0.1
BA.4/5	4.5 ± 1.3	4.4 ± 1.8	3.6 ± 1.8	3.5 ± 1.0	1.2 ± 0.4
BA.2.75	6.0 ± 1.0	3.5 ± 0.5	2.5 ± 0.4	2.1 ± 0.3	2.0 ± 0.4
BQ.1.1	10.7 ± 1.7	6.7 ± 1.3	3.9 ± 1.4	3.6 ± 1.9	3.6 ± 0.7
XBB.1	4.1 ± 0.9	2.4 ± 0.3	0.1	1.9 ± 0.3	1.7 ± 0.2
XBB.1.5	2.1 ± 0.7	4.3 ± 0.4	2.7 ± 0.2	2.7 ± 0.4	2.1 ± 0.4
XBB.1.16	2.6 ± 0.6	1.6 ± 0.4	2.0 ± 0.3	1.9 ± 0.3	1.6 ± 0.4
XBB.1.5.10	977.2 ± 246.8	10.6 ± 2.8	15.4 ± 5.6	11.3 ± 3.4	11.0 2.6
EG.5.1	1142 ± 461.0	7.2 ± 1.6	14.2 ± 5.0	9.2 ± 2.9	8.5 ± 2.2
BA.2.86	3.1 ± 0.6	1.0 ± 0.4	1.0 ± 0.3	1.2 ± 0.3	1.2 ± 0.2
JN.1	87.3 ± 15.4	n.t.	n.t.	n.t.	2 ± 0^*^
JN.1.16	>9000	n.t.	n.t.	n.t.	8551 ± 1502^*^
KP.2	n.t.	n.t.	n.t.	n.t.	37 ± 9^*^
KP.3	>9000^*^	n.t.	n.t.	n.t.	7165 ± 1624^*^

*Assays run at the University of Oxford; EC_50_ values and SDs are calculated from the mean of one experiment, performed in duplicate.

**Table 4.** Antiviral neutralization activity of AZD3152 derivative mAbs against authentic SARS-CoV-2 virus. EC_50_ values and SDs are calculated from the mean of two independent experiments, each in duplicate.

SARS-CoV-2 variants	SARS-CoV-2 neutralization EC_50_ (ng/ml)			
	3152-1070	3152-1100	3152-1132	3152-1142
D614G	28 ± 7	25 ± 4	16 ± 6	26 ± 10
Alpha	10 ± 0	11 ± 1	17 ± 4	6 ± 0
Delta	13 ± 2	11 ± 0	11 ± 0	12 ± 1
BA.1	4 ± 0	6 ± 0	3 ± 0	2 ± 0
BA.1.1	4 ± 1	5 ± 0	2 ± 0	3 ± 0
BA.2	12 ± 2	7 ± 0	3 ± 0	3 ± 1
BA.2.12.1	8 ± 0	5 ± 0	2 ± 0	2 ± 0
BA.5	9 ± 1	2 ± 0	2 ± 0	3 ± 1
BA.2.75	6 ± 1	5 ± 1	5 ± 0	4 ± 1
BQ.1.1	14 ± 0	13 ± 3	11 ± 1	7 ± 1
XBB.1.5	9 ± 0	4 ± 0	4 ± 0	3 ± 0

More recently, a saltation lineage emerged, giving rise to a set of distinct variants (table S1). Saltation events occur far less frequently than gradual adaptive evolution and are characterized by large multi-mutational “jumps” in evolution. Some of these latest variants lacked the F456L mutation, such as BA.2.86 and JN.1 that respectively contain 12 and 13 RBD changes compared to XBB.1.5. In contrast, F456L was present in other variants, including JN.1.16, KP.2, and KP.3, with 14, 13, and 15 RBD mutations, respectively. As expected, AZD3152 lost in vitro activity against the F456L-containing variants (e.g., JN.1.16 and KP.3, EC_50_ > 9000 ng/ml). Our 3152-1142 potently neutralized JN.1, retained strong neutralization against KP.2 (EC_50_, 37 ng/ml), but also lost activity against JN.1.16 and KP.3 (EC_50_, 8551 and 7165 ng/ml, respectively). These results indicate that our approach can be very effective in preemptively optimizing an antibody to future incremental evolution but less effective against the large multi-mutational evolutionary jumps associated with saltation events.

Using biolayer interferometry (BLI) to compare affinities (section S1.6), we further confirmed the improvement of 3152-1142 in RBD binding (table S9 and fig. S6). The affinity of 3152-1142 to XBB.1.5 RBD is approximately 20-fold greater than that of AZD3152 [dissociation constant (*K*_d_) of 96 pM versus 2 nM]. For XBB.1.5+F456L, the *K*_d_ is 17 nM for 3152-1142 and undetectable for AZD3152. Last, neither mAb binds to the KP.3 RBD, confirming our neutralization data (Table 3). The overall consistency between the neutralization and BLI results suggests that the gain in potency for 3152-1142 can be attributed to its stronger binding to the RBD.

### Structural basis for optimized binding

**Fig. 4.**
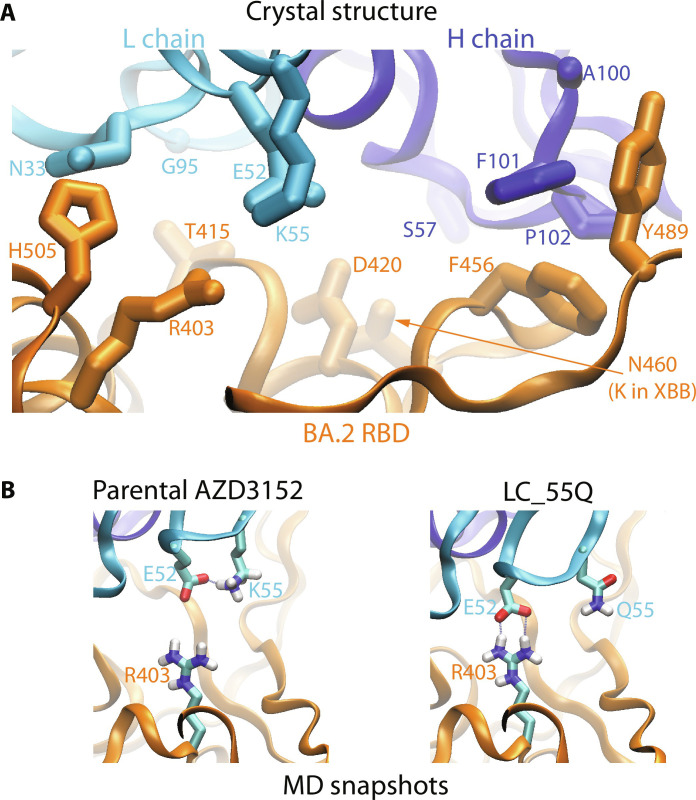
Structural insight into antigen-antibody interactions. (**A**) Important interface residues in the crystal structure (*15*) of AZD3152 bound to BA.2 RBD. (**B**) Snapshots taken from MD simulations of the parental AZD3152 and of its single-point derivative (LC_K55Q), respectively. The side chains of R403 in the RBD and E52 and K55/Q55 in the L chain of the antibody are shown. A salt bridge R403-E52 between the antigen and antibody was formed in the LC_K55Q simulation.

DMS revealed that nearly all substitutions of F456 abolish or reduce AZD3152 binding (Fig. 1), including F456L, despite the fact that it maintains a hydrophobic residue at this position. However, the F456L mutation has a variable effect depending on the specific SARS-CoV-2 background. For example, there was no effect on AZD3152 neutralization when F456L was introduced in the D614G background (with identical RBD sequence to the Wuhan strain) but an approximately 500- and >7000-fold reduction in neutralization for BA.2 and XBB.1.5, respectively (table S10). Our FEP calculations predicted ∆∆*G* of 3.2 ± 0.9 kcal/mol and 4.0 ± 0.5 kcal/mol for the F456L mutation on BA.2 and XBB.1.5 RBDs, respectively, corresponding to substantially weakened AZD3152 binding in both cases, which is consistent with the in vitro results for these two backgrounds. As detailed in section S2.1, our affinity prediction was based on a prereleased crystal structure (PDB code: 8SUO) (*15*). The crystal structure (Fig. 4A) and MD simulations show that F456 is in close contact with F101 and P102 on the HC of AZD3152 Fab. F456 and HC_F101 do not have their phenyl rings directly stacked on top of each other, but F456 does have good shape complementarity with the paratope, which might partially account for the favorability of F456 relative to other substitutions. Given the subtlety of the F456L effects, our top AZD3152 derivatives strategically avoid direct intervention at this position and optimize interactions elsewhere in the binding interface such that improved binding can be achieved for RBDs with either F456 or L456.

The top candidate, 3152-1142, features five amino acid substitutions: S57E and A100E on the HC and N33D, K55Q, and G95H on the LC. Mechanistic implications of these amino acid substitutions can be inferred from the crystal structure (Fig. 4A) and MD simulations (Fig. 4B and fig. S7). Specifically, HC_S57 in AZD3152 is not making interfacial contact with the antigen, but the substituted HC_E57 in 3152-1142 formed a salt bridge with K460 on the XBB.1.5 RBD in our simulation (fig. S7A). Similarly, HC_A100E could introduce favorable additional intermolecular contacts, effectively expanding the epitope; MD simulation showed an H-bond between HC_E100 and RBD Y489 (fig. S7B). In addition, LC_N33D might strengthen interactions with H505 on the RBD; in simulation, the LC_D33 in 3152-1142 formed H-bond with N405 or H505 (fig. S7C). The effect of LC_K55Q substitution is more nuanced. The LC_K55 in AZD3152 is proximal to R403 on the RBD, an unfavorable electrostatic interaction. Also, LC_K55 makes an intra-LC salt bridge with LC_E52 (Fig. 4B), reducing the strength of intermolecular interactions from these residues. The LC_K55Q substitution disrupts the intra-LC salt bridge and allows LC_E52 to form a salt bridge with RBD R403 (Fig. 4B), thus enhancing antigen-antibody attraction. Last, the LC_G95H substitution increases the buried surface area of the Fab; in the 3152-1142 simulation, the LC_H95 side chain was flexible, occasionally interacting with RBD T415 or nearby backbone atoms (fig. S7D).

Each of the individual substitutions in 3152-1142 was present in some of the candidates (as single- or double-point derivative) from our first design iteration, but those candidates did not sufficiently restore the binding to XBB.1.5+F456L. The combined substitutions in 3152-1142 appear to be complementary, resulting in substantial collective enhancement. Notably, while 3152-1142 is the best performing design overall, several other candidates also demonstrated highly favorable attributes and differ from 3152-1142 by only one or two substitutions.

Although the inferred Fab-RBD interactions described here are qualitatively consistent with the neutralization and BLI binding experiments, we note that they are still speculations that remain to be further verified. Definitive conclusion will require solving complex structures for 3152-1142 in future studies.

### 3152-1142 introduces no additional vulnerability

To fully characterize the breath of 3152-1142, we repeated DMS on the XBB.1.5 RBD yeast-displayed library. 3152-1142 demonstrated improved binding across most of D420 and F456 substitutions (Fig. 3E, and fig. S8, and data file S2), confirming our earlier monoclonal yeast-binding results (Fig. 3D). On the other hand, for some residues in the RBD 435-452 region where the escape potential is relatively low, 3152-1142 has higher scores than AZD3152 does (Fig. 3E). However, given that the region is not at the binding interface and that some involved residues are buried in the protein interior, the apparent escape scores would likely arise from changes in the RBD folding rather than antibody binding, thus regarded as less relevant. In conclusion, we observed a noticeable improvement in escape scores for positions 420 and 456 and did not detect additional major liability to 3152-1142 at other RBD positions. This finding supports our structural analysis suggesting that the improvement in binding is distributed across the introduced amino acid substitutions, potentially offering greater tolerance of single RBD mutations.

## DISCUSSION

The continuing evolution of SARS-CoV-2 poses a challenge to the clinical development of mAb therapies, which have been partially or fully escaped by RBD mutations. Antibody optimization has been achieved by a variety of methods such as site-directed mutagenesis (*21*) and directed evolution (*22*). Complementary to experimental techniques, computational redesign can also successfully rescue a clinical antibody from escape, as recently demonstrated for COV2-2130 (*11*). In this study, we adopted a similar approach (*11*) for antibody redesign but incorporated DMS for identifying liabilities and vastly increased the number of variants, both known and predicted, to optimize against. We carried out two design cycles along with experimental validation to enhance AZD3152. Our lead candidate from this computational design, 3152-1142, achieves much higher breadth than AZD3152, without introducing additional escape vulnerabilities. We hypothesize that by introducing multiple mutations throughout the antibody-antigen interface that broadly improve binding, the redesigned antibody is less vulnerable to RBD mutations than AZD3152, allowing it to maintain high potency and breadth.

Further, 3152-1142 exhibited ~100-fold enhancements in the neutralization of the XBB.1.5.10 and EG.5.1 variants with the F456L mutation, which emerged during the course of this study and escaped AZD3152. 3152-1142 restored potency to the escape variants while maintaining or improving potency against previous variants (Table 3). Moreover, 3152-1142 demonstrated favorable developability properties (Table 2). Overall, our optimization strategy yielded a superior mAb in terms of breadth, potency, and developability. As the F456L mutation was not previously seen in earlier VOCs, our antibody design approach both anticipated and preemptively mitigated viral escape for AZD3152.

It is often assumed that mutational effects from individual antibody/antigen positions are approximately independent of each other such that their combined effects can be additive. However, recent systematic studies (*23*, *24*) revealed epistasis for SARS-CoV-2, where the escape potential for a given RBD mutation could increase as the variant background evolves, thus casting doubt on the independence of mutational effects. Such epistasis is consistent with our findings here that the effect of the F456L mutation on antibody escape depends on the variant background, as was observed for the AZD3152 binding to the ancestral and early Omicron variants (table S10) as well as the 3152-1142 binding to the BA.2.86 sublineages (Table 3). Epistasis implies certain allosteric effects at the structural level, where mutations on some residues may subtly change the RBD conformation and thereby regulate the mutational effects of other residues. As XBB.1.5 was used for our computational optimization, 3152-1142 fully recovered activity lost by AZD3152 against F456L in this background. However, BA.2.86 and its sublineages, which emerged more than 1 year after completing our first design iteration, differ substantially from XBB.1.5, and 3152-1142 was not as successful on these backgrounds. To further enhance preemptive mitigation, we should aim to either better predict future variant backgrounds or incorporate much broader sets of RBD sequences in the optimization.

Under evolutionary pressure, SARS-CoV-2 will continue to produce variants that evade vaccines and antibodies (*25*). Because our computational redesign was initiated solely on the basis of our understanding of potential escape vulnerabilities following analysis of DMS data, before the emergence of F456L-containing variants, this work demonstrates that our approach can be used to anticipate and mitigate future antibody escape liabilities to some extent while maintaining efficacy to existing and historical variants. One limitation of our work is the challenge of mitigating against unpredicted large antigenic shift, such as the multi-mutational evolutionary jumps that occur as saltation events. In our case here, the appearance of the BA.2.86 saltation lineage, which gave rise to the JN and KP sublineages, carried with it 12 amino acid changes in the RBD compared to XBB.1.5. In this context, the F456L mutation resulted in a reduction of 3152-1142 activity, highlighting the difficulty of anticipating epistatic effects as discussed earlier. Therefore, our approach may need to be reused when saltation lineages such as BA.1 or BA.2.86 emerge. Most SARS-CoV-2 antibodies have a “shelf life” before being evaded by virus mutations, with none guaranteed to remain effective against all possible future variants. Albeit no guarantee of indefinite robustness to viral evolution, our approach nonetheless demonstrates an ability to extend the shelf life with a combination of DMS and preemptive computational optimization.

## MATERIALS AND METHODS

### Study design

Experiments in this study include yeast DMS, flow cytometry, BLI binding assays, RBD-ACE2–blocking assays, neutralization assays with pseudovirus and authentic virus, and a set of developability assays. Experimental details are provided in section S1.

Computational methods consist of the following components: (i) structure-based predictions of relative binding affinity using the Rosetta Flex, SFE, PMF, and FEP tools; (ii) first design iteration with multiobjective optimization techniques; and (iii) second design iteration with the “select, combine, and diversify” strategy. Details of these methods are provided in section S2.

### Statistical analysis

Individual-level data for experiments where *n* < 20 are presented in data file S2. Neutralization data were graphed and analyzed in GraphPad Prism (GraphPad Software Inc.; version 9.0.0 or later); neutralization curves were fit using nonlinear regressions; EC_50_ values were generated in Prism.

## Supplementary Materials

### The PDF file includes:

The LLNL GUIDE Consortium Supplementary Text Figs. S1 to S8 Tables S1 to S10 Legends for data S1 and S2 References
