# Supplementary material for: Preemptive optimization of a clinical antibody for broad neutralization of SARS-CoV-2 variants and robustness against viral escape
Source: Sci Adv. 2025 Mar 28;11(13):eadu0718. doi: 10.1126/sciadv.adu0718 (PMC11952088; doi:10.1126/sciadv.adu0718)
Supplement: Supplementary file 1 — The LLNL GUIDE Consortium Supplementary Text Figs. S1 to S8 Tables S1 to S10 Legends for data S1 and S2 References [file sciadv.adu0718_sm.pdf]

Science Advances
AAAS

Supplementary Materials for

Preemptive optimization of a clinical antibody for broad neutralization of SARS-CoV-2 variants and robustness against viral escape

Fangqiang Zhu *et al.*

Corresponding author: Daniel M. Faissol, faissol1@llnl.gov; Joseph R. Francica, joe.francica@astrazeneca.com

Sci. Adv. **11**, eadu0718 (2025)
DOI: 10.1126/sciadv.adu0718

The PDF file includes:

The LLNL GUIDE Consortium
Supplementary Text
Figs. S1 to S8
Tables S1 to S10
Legends for data S1 and S2
References

Other Supplementary Material for this manuscript includes the following:

Data S1 and S2

The LLNL Generative Unconstrained Intelligent Drug Engineering (GUIDE) consortium

In addition to GUIDE members who are authors (Fangqiang Zhu, Conor F. Hayes, Andre R. Goncalves, John W. Goforth, Adam T. Zemla, Edmond Y. Lau, Thomas A. Desautels, Kathryn T. Arrildt, Thomas W. Bates, Drew Bennett, Simone Conti, Brent W. Segelke, Samantha Kaul, Emilia A. Grzesiak, Mikel Landajuela, Felipe Leno da Silva, Shankar Sundaram, and Daniel M. Faissol), the following GUIDE members supported operations underlying the antibody designs reported here: Olajide Alawode, Teneile Alfaro, Aram Avila-Herrera, Brian Bennion, Thomas Bunt, Rudra Chakraborty, Matt Coleman, Nicole Collette, Zach Cosenza, Ana Paula De Oliveira Sales, Jie Deng, Aneesha Devulapally, Jeff Drocco, Becky Haluska, Fareeh Kanwal Malik, Piyush Karande, Sun Kyung Kim, Dan Kirshner, Xander Ladd, Doris Lam, Sabrina Lopez, Summer McCloy, Tavish McDonald, Kevin Mcloughlin, Shakiba Nikfarjam, Christine Ocampo, Daniel Osei-Koffuor, Kim Phan, Hiranmaya Ranganathan, Tiffany Reck, Dante Ricci, Patricia Rines, Bonnee Rubinfeld, Ed Saada, Michael Sanchez, Sarah Sandholtz, Megan Shelby, Mary Silva, Cheryl Strout, Jayram Thathachar, Uttara Tipnis, Gary Trubl, Camilo Valdes, Dillon Vannest, Denis Vashchenko, Yaqing Wang, Nekesa Wanjala, Nick Watkins, Dina Weilhammer, Tracy Weisenberger, Dawn Whalen, Jessica Wollard, Boya Zhang, Teija Isotalo, Rachele Scott, John Hernandez, and Ryann Swale.

Supplementary Text

S1. Experimental methods

S1.1 Yeast deep mutagenesis scanning and visualization

S1.1.1 Deep mutational scanning (DMS) library construction

XBB.1.5 RBD sequence was codon optimized for expression in yeast and fused to C-terminal of yeast Aga2p and cloned into in-house developed yeast display vector. 4 unique restriction enzymes cutting sites were introduced to the coding region of RBD, roughly divided RBD in 3 sections to facilitating the cloning process: Nde1, Ale1, BstB1 and Xho1. Single stranded DNA were synthesized as 3 separate oPools Oligo Pools by IDT, each oPools covered roughly one third of RBD. The design of the libraries was that in each oligo, one and only one amino acid (AA) in each RBD positions were replaced by NNK codons, each of the 201 AA was covered by one set of NNK containing oligos. The 3 oPools were converted to double stranded DNA by one cycle of PCR using corresponding reverse primers. Parental XBB.1.5 RBD yeast display vector was digested with combination of two restriction enzymes to remove the region to be replaced by double stranded oPools DNA: Nde1+Ale1, Ale1+BstB1, BstB1+Xho1. Digested and gel-purified yeast display vectors were mixed with corresponding double stranded oPools DNA, incubated with NEB HiFi reaction mix at 50C for 15 minutes in three separate reactions. The reaction mixes were purified by NEB Monarch PCR cleanup kit, then electroporated into NEB5 alpha cells. Aliqouts were plated for titre calculation and the rest plated on LB Agar with 100ug/ml carbenicillin. The number of transformants were > 4 $X10^5$, at least hundred fold higher than the predicted libraries complexity. Bacteria was scrapped from agar surface, and plasmid was extracted. 10ug of library DNA (3 libraries pooled together) was digested with Swa1 to linearize DNA for transformation into the EBY100 strain of *S. cerevisiae*. A PCR fragment overlapping with the Swa1 site on the vector backbone was co-electroporated to increase transformation efficiency. The overall yeast transformants was more than 10^6. Overnight culture of the XBB.1.5 DMS library was collected and aliqouts frozen at -80C.

DMS library was inoculated from frozen storage into 5ml of CM Glucose Broth minus Tryptophan (Teknova C8130), shake at 250 rpm, 30C for overnight or two days until culture reach saturation phase (around OD 5-7). From this starter culture, density was adjusted to OD 0.5 and inoculated into 9:1 mix of 5 ml CM Galactose Broth minus Tryptophan (Teknova C9115) and CM Glucose Broth minus Tryptophan (Teknova C8130) for induction of RBD expression at 20C overnight.

S1.1.2 Cell staining and sorting

5 ml of yeast was centrifuged for 3 min at 3500g and resuspended into 500ul FACS buffer (1 X phosphate buffered saline, 0.1% bovine serum albumin). Candidate antibody at 1 nM or biotinylated human ACE2 (Acrobiosystems AC2-H82E6) at 5 nM final concentration and anti-myc tag antibody 9B11 (Cell Signaling Technology 2276S) at 1:1,000 dilution was incubated with yeast library at room temperature for 1 hour. The cells were washed two times with FACS buffer (1 X phosphate buffered saline, 0.1% bovine serum albumin) and stained with the appropriate secondary detection reagents: for human IgG staining: 1:500 Alexa 488 conjugated goat anti-human Fc Jackson ImmunoResearch 109-545-008 and 1:200 APC conjugated goat anti-mouse Jackson ImmunoResearch 115-136-146); for human ACE2 staining: 1:2,000 streptavidin conjugated with APC (BioLegend 405243) and 1:1,000 Alexa 488 conjugated goat anti-mouse

(Cell Signaling Technology 4408S). After a 30-minute incubation at room temperature, the yeast cells were washed two times and then resuspended with 5 ml FACS buffer and kept on ice in the dark until analysis on the Sony cells sorter.

S1.1.3 NGS procedure for escape mutants

Roughly 20000 RBD expression positive, mAb binding negative cells were collected into 2 ml of CM Glucose Broth minus Tryptophan in a 15 ml collection tubes. The cells were centrifuged at 3500g for 3 minutes to ensure cells were not attached to side wall. The cells were cultured overnight (or up to 2 days) until culture become cloudy. The culture was centrifuged to isolate the yeast pellet and DNA was extracted by Zymoprep Yeast Plasmid MiniPrep II (Zymo Research) according to manufacturer protocol. For an unsorted RBD library control, DNA was extracted from the yeast culture before analysis via the Sony cell sorter. The RBD coding region was amplified by PCR primers, gel-purified, and quality checked by Qubit dsDNA HS assay (ThermoFisher) and Tapestation high sensitivity D1000 assay (Agilent). Libraries were then generated using Ultra II DNA FS kit (New England Biolabs) following standard manufacturer protocol. Sequencing was performed using 2X250 bp protocol on the Illumina MiSeq. We validated the library by confirming that 99% of expected codons were identified at each position.

S1.1.4 Bioinformatics pipeline for DMS

The saturation mutagenesis strategy described above resulted in 4,020 unique, and 3,819 valid RBD sequences (201 RBD sequences had a premature stop codon). Due to codon degeneracy, it was possible that some expressed RBD sequences were present up to 3 times in the construct pool. Our analysis pipeline included the following steps:

1) Database construction based on saturation mutagenesis strategy above, and separation of all RBD sequences into mutant and wildtype based on the XBB.1.5
2) Deduplication of sequencing library to retain only unique reads
3) After primer exclusion, perform BLAST to identify putative mutant sequences by removing all sequences with exact matches (e-value=1e-10 & percent identity=100) to the wildtype RBD AA sequence.
    a. Furthermore, reads are retained if their entire length aligns with the subject sequence (RBD). If only part of the read aligns, then we required that the first alignment position in the subject sequence is at the start or end (reverse orientation) of the query sequence (RBD)
4) For the remaining sequences, perform BLAST against reference database of mutant sequences to identify exact matches (using the same criteria as above) and generate the count matrix of reads per variant per sample normalized to copies per million.
    a. Remove, singleton hits, split read alignments and mis-captured sequences (sequences with mutations outside of region where known variation occurs)
5) Finally, remove mutants with low representation in the pre-sort sample and calculate the final escape score per mutation as a function of the mean reverse rank order of the fold change (ratio) and fold difference for the post-sort vs. pre-sort conditions.

    Escape score = 1 – {rank – min(rank)/max(rank) – min(rank)},

    where rank = average (fold-change rank, fold difference rank)

S1.2 Yeast monoclonal binding by flow

Yeast expressing RBD with substitutions at positions 420 and 456 were cultured in CM Glucose Broth minus Tryptophan (Teknova C8130), shake at 250 rpm, 30C for overnight. On the next day, the cultures were inoculated 1/15 into 9:1 mix of CM Galactose Broth minus Tryptophan (Teknova C9115) and CM Glucose Broth minus Tryptophan (Teknova C8130) for induction of RBD expression at 20C overnight. 5 ul of yeast was added to 150ul of FACS buffer in V-bottom 96 well assay plate (Costar 3897) and spun down at 3500g for 3 minutes. The supernatant was discarded, and 50ul of FACS buffer was added with 1nM tested anti-RBD antibody and 1:1000 anti-myc antibody (clone 9B11, Cell Signaling Technology), then incubated at room temperature for 60 minutes. Cells were washed twice with FACS buffer. Secondary detection reagents were added and incubated for 30 minutes at room temperature, protected from light. Two detection combinations were used: 1. APC-conjugated goat anti-human Fc (1:200 Jackson Immunoresearch laboratory 109-135-098) and Alexa488-conjugated anti-mouse (1:2000 Cell Signaling Technology 4408S); or 2. BV421-conjugated goat anti-human Fc (1:500 Jackson Immunoresearch laboratory 109-675-098) and APC-conjugated goat anti-mouse (1:1000 Jackson Immunoresearch laboratory 115-136-146). After secondary antibodies staining, the yeast was washed once with FACS buffer and resuspended into 100 ul FACS buffer.

For co-culture assays to measure parallel binding to wild-type RBD and its mutants, we first constructed a yeast strain expressing GFP and displaying the XBB.1.5 RBD simultaneously. This was achieved using the yeast bi-directional GAL10/GAL1 promoter, where RBD expression was under control of GAL1 promoter and GFP protein expression was driven by the GAL10 promoter. Prior to staining, GFP expressing parental XBB.1.5 yeast was mixed with non-GFP expressing XBB.1.5 mutants at 1:1 ratio and stained as above. Antibody binding was via secondary detection BV421-conjugated goat anti-human Fc (1:500 Jackson Immunoresearch laboratory 109-675-098) and RBD detection as above via anti-myc secondary followed by APC-conjugated goat anti-mouse tertiary (1:1000 Jackson Immunoresearch laboratory 115-136-146) and only yeast displaying RBD were gated for analysis. Flow cytometry was performed via a Sartorius iQue3 and analyzed by Sartorius Forecyt software.

S1.3 Antibody production

The variable heavy and light sequences from candidate antibodies were synthesized as DNA eBlocks (IDT) and cloned using recombination (NEBuilder, NEB) into an IgG expression vector containing modifications within the Fragment crystallizable region (Fc) for half-life extension (YTE) and attenuation of effector function (Triple Mutant, TM). Small-scale IgG production was accomplished by transient transfection of HEK-293 cells with plasmid DNA using 293fectin (ThermoFisher). Cells were grown in deep-well plates at 37°C, 8% CO_2, 80% Humidity, and fed on day 3 with Freestyle media (ThermoFisher). Supernatants containing secreted IgG were harvested 5-7 days post-transfection. For the second iteration designs, we also conducted high throughput protein purification using MagSepharose PrismA (Cytiva Cat # 17550001) with Hamilton Microlab STAR Liquid Handler.

S1.4 Antibody-RBD binding by ELISA

MaxiSorp 384-well plates (Nunc) were coated overnight at 4°C with 25 µL antigen per well at 1µg/mL. SARS-CoV-2 antigens used were as follows: B.Q.1.1-RBD (AcroBiosystems, cat#SPD-C5240), Wuhan-RBD (made in-house), XBB.1.5-RBD (AcroBiosystems cat#SPD-C5242) and negative control antigen human SLAMF7 ECD (made in-house). Wells were then incubated with blocking buffer (4% BSA in PBS with 0.1% Tween20) for 1 hr at room temperature. Plates were washed with PBS+0.1% Tween20 and 25 µL of 1:10 antibody diluted in Blocking buffer were added to wells. After 1 hr incubation and subsequent plate wash, 25 µL of 1:10,000 Peroxidase conjugated anti-Human Fc-gamma-HRP (Jackson ImmunoResearch) in blocking buffer was added to all wells for 30 min. Following a final wash, TMB SureBlue (KPL) was added for colorimetric development for 5 min and the reaction stopped with equal volume of 1M HCl. Plates were read at 450nm on an EnVision 2104 plate reader (Perkin-Elmer).

S1.5 RBD-ACE2 blocking assay

Biotinylated XBB.1 RBD-Avi-tag protein (AcroBiosystems) was coupled to six-micron streptavidin-conjugated beads (SpheroTech). Beads were washed with RoboSep buffer (StemCell Technologies) and stored as 5% suspension in RoboSep buffer at 4C°. To assess ACE2 blocking activity, RBD-coupled beads were diluted to 0.05% with RoboSep buffer and dispensed at 10 uL per well into the wells of V-bottom 96-well plates (Costar). Tested antibodies were diluted to 40 ug/mL in a RoboSep buffer, and 10 uL of each antibody was added the beads to the final mAb concentration of 20 ug/mL followed by 15 min incubation on a plate shaker at 200 rpm. Twenty uL of Alexa Fluor 647-labeled hACE2 (R&D) was added to each well for a final concentration of 1 ug/mL ACE2 followed by brief shaking of the plate and 20 min incubation at ambient temperature. Control for the background from no ACE2 binding included duplicate wells with mock (no RBD coupling) beads plus Alexa Fluor 647-labeled hACE2. Control for maximal ACE2 binding without antibody-blocking activity included binding of Alexa Fluor 647-labeled hACE2 to the RBD-coupled beads in the presence of irrelevant mAb K347 which is not reactive to XBB.1 RBD. Fluorescence from bound Alexa Fluor 647-labeled hACE2 was assessed using an IntelliCyt iQuePlus Screener flow cytometer (Sartorius). The percent of ACE2 binding inhibition was calculated from the median fluorescence intensity in each well relative to the maximal ACE2 binding from the control minus background using Forecyt software (Sartorius).

S1.6 RBD binding kinetics by biolayer interferometry (BLI)

BLI was performed using an Octet RED96 instrument (ForteBio; Pall Life Sciences). Purified IgG was first captured onto AHC biosensor tips (loading 200 s). The IgG-loaded biosensor tips then were submerged in binding buffer (kinetics buffer (Sartorius #18–1105) in PBS) containing serial dilutions (for full IgG multi-point kinetics) of SARS-CoV-2 XBB.1.5 RBD, XBB.1.5+F456L RBD (internally produced), and KP.3 RBD (SinoBiological #40592-V49H18-B) protein. We used a minimum response of 0.20 nm to determine fits using a 1:1 model.

S1.7 SARS-CoV-2 pseudovirus neutralization assay

To generate severe acute respiratory syndrome coronavirus 2 (SARS-CoV-2) pseudoviruses, we implemented a third-generation human immunodeficiency virus-based lentiviral vector system. Briefly, we co-transfected freestyle 293X cells with the following four plasmids: (i) pAZRev (lentiviral packaging plasmid); (ii) pPACKH1-Gag (lentiviral packaging plasmid); (iii) pESRC-CMV-Luc2p-EF1Puro (luciferase reporter plasmid); (iv) pCAGG-Sdl19Gen (SARS-CoV-2 spike variant with C-terminus 19 AA deletion-expressing plasmid). Table S1 listed the spike amino acid mutations that are different from the ancestral Wuhan strain. The viral supernatant was harvested 48 hours later. Cell debris was removed by low-speed centrifugation and the supernatant was passed through a 0.45-µM filter unit. To prepare pseudoviruses bearing the spike protein of the D614G, D614G +F456L, Alpha, Beta, Delta or Gamma variants, the supernatant was concentrated 100-fold by ultracentrifugation at 46,677 x g with an SW32 Ti rotor for 2 hours at 4°C. To prepare pseudoviruses bearing the spike from Omicron BA.1, BA.1.1, BA.2, BA.2 + F456L, BA2.12.1, BA.4/5, BA.4/5 + F456L, BA.2.75, BQ.1.1, XBB.1, XBB.1.5, XBB.1.5 + F456L, XBB.1.16, XBB.1.5.10, EG.5.1 or BA.2.86, the clarified supernatants were loaded on the top of 10% sucrose cushion at 4:1 v/v ratio of virus:sucrose and centrifuge at 10,000 x g for 4 hours at 4°C. Serial dilutions of mAbs were prepared in a 384-well microtiter plate and pre-incubated with pseudovirus for 60 minutes at 37°C, to which Ad293 cells that stably express ACE2 were added. The plate was returned to the 37°C incubator for 48 hours and luciferase activity was measured on an EnVision 2105 Multimode Plate Reader (PerkinElmer) using the Bright Glo Luciferase Assay System (Promega) according to the manufacturer's recommendations. Half-maximal effective concentrations (EC_{50}) were determined from nonlinear regression analyses.

S1.8 SARS-CoV-2 focus reduction neutralization test (FRNT)

The neutralization potency of each mAb against pic SARS-CoV-2 was measured using a FRNT as previously described (*26*). All work with infectious SARS-CoV-2 was approved by the University of Oxford Institutional Biosafety Committees and conducted in approved biosafety level 3 facilities, using appropriate powered air purifying respirators and personal protective equipment. SARS-CoV-2 strains D614G, Alpha, Delta, and Omicron (BA.1, BA1.1, BA.2, BA.2.12.1, and BA.5) were isolated from patient swabs. The spike amino acid mutations that are different from the ancestral Wuhan strain are listed in Table S1. Viruses were passaged in Vero E6/TMPRSS2 cells (Vero E6 cells stably expressing transmembrane serine protease 2; NIBSC, reference number, 100978) and titrated by plaque assay on Vero cells. Serial dilutions of mAbs were mixed with SARS-CoV-2 strains (D614G, Alpha, Delta, Omicron [BA.1, BA1.1, BA.2, BA.2.12.1 or BA.5]) and incubated at 37°C for 1 hour. The mAb–virus mixtures were then transferred to 96-well, cell culture–treated, flat-bottom microplates containing confluent Vero cell monolayers in duplicate and incubated at 37°C for an additional 2 hours, followed by the addition of 1.5% semi-solid carboxymethyl cellulose overlay medium to each well to limit virus diffusion. A focus forming assay was then performed by staining Vero cells with 2 µg/mL human anti-nucleocapsid protein (NP) mAb (catalogue number, mAb206) as primary antibody, followed by peroxidase-conjugated goat anti-human IgG diluted 1:5000 (Sigma-Aldrich). Finally, TrueBlue Peroxidase Substrate was added to each well to visualize the foci (infected cells). Virus-infected cell foci were counted on the classic AID EliSpot reader using AID ELISpot software (Immunospot). The percentage of focus reduction was calculated relative to the virus alone control.

EC_{50} values were determined from non-linear regression analysis using GraphPad Prism version 9.0.0. The average EC_{50} value for each mAb was determined from two independent experiments.

S1.9 NIP228 and "NSB mAb" control antibodies

NIP228 is a monoclonal antibody against 4-hydroxy-3-iodo-5-nitrophenylacetic acid that historically has been shown not to exhibit aggregation/fragmentation propensities under similar heat/photo stress conditions and is used as a negative control in select developability assays. The "NSB mAb" is a monoclonal antibody against an undisclosed target not related to SARS-CoV-2 that shows high BVP scores and plate binding in the Baculovirus ELISA and high HEK293 cell binding in the HEK binding assay and is used as a positive control antibody in these assays. Unless otherwise stated, both control antibodies are on a WT human IgG1 Fc. Both control antibodies were discovered and produced in-house.

S1.10 Baculovirus ELISA

BVP ELISA was performed essentially as described elsewhere, with some modifications. Briefly, a 1% BV suspension in 50 mM sodium carbonate buffer (pH 9.6) was used to coat half of 96-well ELISA plates (Nunc Maxisorp) overnight at 4°C, while the second half of the ELISA plates was left uncoated to test the antibodies for plate binding. All following steps were performed at room temperature. The next day the wells were washed with Dulbecco's PBS (DPBS) and then incubated with blocking buffer (Dulbecco's PBS with 0.5% BSA) for 1 h, followed by three washes with DPBS. Next, test antibodies at 100 nM and 10 nM in blocking buffer were added to both the BVP coated and uncoated wells and incubated for 1 h, followed by three washes with DPBS. Next, goat anti-human IgG-horseradish peroxidase (HRP) secondary antibodies (1:5000 dilution, Sigma-Aldrich #A0170) in blocking buffer were added to the wells and incubated for 1 h, followed by three washes with DPBS. Finally, 3,3',5,5'-tetramethylbenzidine substrate (SeraCare #5120-0075) was added to each well and incubated for 2 minutes. The reactions were stopped by adding an equal volume of 0.2 M sulfuric acid to each well. The absorbance was read at 450 nm. BVP score and plate binding were determined by normalizing absorbance to control wells with no test antibody.

S1.11 HEK binding assay

Nonspecific HEK cell binding was measured using a Mirrorball Fluorescence Cytometer (SPT Labtech). First, 10 µL of Alexa Fluor 647 goat anti-human IgG (H + L) antibody (Invitrogen #A-21445) diluted to 16 nM in Mirrorball buffer (Hanks' Balanced Salt solution with 0.5% BSA) was added to wells of a 384-well, clear bottom plate. Next, 10 µL of test antibody serially diluted in Mirrorball buffer was added to the wells. Finally, 20 µL of HEK293f cells diluted to 250,000 cells/mL in Mirrorball buffer was added to the wells. The plate was incubated at room temperature for 2 h, and the fluorescence of each well was measured using the Mirrorball Fluorescence Cytometer.

S1.12 AC-SINS

AC-SINS was performed essentially as described elsewhere (*27*), with some modifications. Briefly, both whole goat IgG (Jackson ImmunoResearch #005-000-003) (noncapture) and polyclonal goat anti-human IgG Fc (Jackson ImmunoResearch #109-005-098) (capture) antibodies were dialyzed into 20 mM potassium acetate (pH 4.3) buffer, and then conjugated to 20 nm gold nanoparticles (Innova Biosciences #3201-0100) at a 3:2 ratio of capture:noncapture antibodies. Antibodies were incubated with gold nanoparticles at a 9:1 ratio for 1 h at room temperature, and then blocked by the addition of 0.1 µM poly-(ethylene glycol) methyl ether thiol (2000 MW, Sigma-Aldrich #729140) for 1 h. The coated and blocked nanoparticles were concentrated 12.5-fold by centrifugation and stored at 4°C. To assess self-association, 5 µL of nanoparticles were mixed with 45 µL of purified antibody at 50 ug/mL in PBS, pH 7.2 or HSA buffer [20 mM histidine, 120 mM sucrose, 80 mM arginine, pH 6] in a 384-well plate. Nanoparticles were mixed with buffer only (no antibody) as a control. Absorbance was measured on a SPECTROstar Nano UV/vis plate reader from 490 to 700 nm. The wavelength of peak absorbance was calculated in the MARS data analysis software and used to determine the wavelength shift compared to the nanoparticle-only control.

S1.13 Accelerated stability heat stress study

For accelerated stability testing, samples were diluted to 1 mg/mL in PBS (pH 7.2) and incubated for 2 weeks at either 4°C or 45°C. Samples were then analyzed by HP-SEC. The monomer, aggregate, and fragment percentages for each sample were calculated based on curve integration using the HPLC ChemStation software (Agilent). The change in monomer, aggregate, and fragment content was calculated from the difference between each sample incubated at 45°C versus 4°C.

S1.14 Photostability assay

For photostability testing, antibodies were formulated at 2.5 mg/mL in PBS (pH 7.2), filled into 1cc Schott glass vials, stoppered/sealed, and placed into an ICH-compliant photostability chamber (Caron Model 6545-2). Samples were exposed to cool white light at 2000 lux. Samples were analyzed by HP-SEC. The change in monomer, aggregate, and fragment content was calculated from the difference between each sample incubated at 45°C versus 4°C.

S2. Computational methods

Computations in this study include binding affinity calculations by various methods and two design iterations for selecting antibody derivatives, as described below.

S2.1 Methods for estimating antibody-antigen binding affinity

For affinity prediction, we used computational tools as described in our recent work (*11*) on COV-2130. In this section, we focus on the details specific to the AZD3152 system in this study. These calculations were based on a pre-released crystal structure featuring the antigen-binding fragment (Fab) of AZD3152 in complex with the BA.2 RBD; the structure was later deposited with PDB code 8SUO after further refinement (*15*).

S2.1.1 Rosetta Flex calculations

Rosetta Flex (*18*) was used to predict binding affinities to XBB and BA2 variants for both single and double-point mutants of AZD3152. We utilized antigen-antibody complex structures from two source models 7xb0 (*28*) and the crystal structure, with XBB and BA2 variants plus 420(A/I/L/R) and 456(D/L/N/R/V) single antigen mutations for a total of 40 complex structures (2 models * 2 parental antigen variants * 10 single point mutations including wildtype.) Using a 7Å interface across 40 complex structures, the union of all residue positions within interfaces was identified. On this interface union, exhaustive single point substitutions (using all AAs except cysteine and proline) were evaluated in Rosetta Flex, 835 mutants in total. Approximately 137,000 double-point mutants were simulated using Rosetta Flex against the XBB (wildtype) CyroEM structure. This batch of 137,000 mutants was selected from the broader set of possible double-point mutants by examining both Rosetta Flex and SFE (see below) single-point results. The 15,000 double-point mutants with the highest predicted binding scores ($\Delta\Delta G$) against XBB were then simulated with Rosetta Flex against other structures to evaluate predicted binding in other variants.

S2.1.2 Structural fluctuation estimation (SFE) calculations

In the SFE approach to estimate binding affinities between mutants of the antibody AZD3152 and the RBD variants BA.2 and XBB we used the same protocols as described in our previous work (*11, 19*). The SFE processing started from selecting two reference structural models of the RBD-FAB co-complexes: 1) a pre-released X-ray structure of the BA.2-AZD3152 co-complex, and 2) a modified model of the BA.2-AZD3152 complex when the RBD is replaced by the X-ray structure of the Omicron BA.2 (PDB id 7xb0, chain B, resolution 2.90 Å). Two additional reference complexes XBB-AZD3152 were constructed using the reference models of BA.2-AZD3152 co-complexes as structural templates.

The reference structural models were then used to construct 14,020 (11,332 single and 2,688 multiple-point) mutant models of RBD-AZD3152 complexes. All models were further processed using standard minimization procedures from Rosetta (using an energy scoring function ref2015), Chimera ("minimize structure" using ff14SB) and GROMACS steepest descent and conjugate gradient methods followed by short MD simulations (1 ns using ff99SB) to collect a set of 30 diverse structure conformations for each of the processed complexes. Each set of structure conformations was further processed to estimate binding affinity changes upon introduced mutations ($\Delta\Delta G$) using Rosetta Flex-ddg protocol (*18*) implemented with the scoring function Talaris14.

In the SFE protocol, we perform "forward" (on models without mutations, i.e. 4 reference models) and "reverse" (on models with mutations, i.e. 14,020 mutated model) calculations. When Rosetta Flex-ddg calculations are completed we remove outliers, average results of the interquartile simulations, and calculate the final $\Delta\Delta G$ as estimated by the formula: $\Delta\Delta G$=($\Delta\Delta G^{\text{forward}}$

- $\Delta\Delta G^{\text{reverse}}$)/2. The resulting $\Delta\Delta G$ value provides an affinity estimate that has been shown to be more reproducible and robust than $\Delta\Delta G$ estimates calculated from just one initial input structure.

For the constructed mutant models the positions to mutate were identified based on residues observed within 7Å interface between AZD3152 and RBD variants. 11,332 single point mutants were created by substituting different amino-acids at each position. Positions and mutations used in the construction of 2,688 multiple-point mutant models were selected by combining 2 to 5 single-point mutants that individually showed possible improvements in estimated binding affinities (low values in calculated $\Delta\Delta G$).

S2.1.3 Potential of mean force (PMF) simulations

PMF simulations (*17*) were performed to predict binding affinity between the antibody AZD3152 and the XBB RBD. The protocols used for the PMF calculations have been detailed previously (*11*). Briefly, the antibody-antigen complex was equilibrated in a cubic box of TIP3P water (*29*). The final structure was pared down to a water shell (*30*) that was 8 Å from any atom in the protein complex. The PMF calculations were performed in eight 1 Å separations between the antibody and antigen. The sampling was performed for 320 ns for each separation. Residues selected for study under this protocol were in the CDRH3 at HC positions F101, P102, Y104, S105, Y109, and Y110. For each residue position, the AA was mutated to all other residues besides cysteine and proline.

S2.1.4 Free energy perturbation (FEP) simulations

FEP (*16*) is a rigorous physics-based method that employs molecular dynamics (MD) simulations to calculate the change in binding affinity due to mutation. The FEP simulations in this study adopted the same protocols as in our previous works (*11, 16*), using the Amber18 software package (*31*) with the ff14SB (*32*) and TIP3P (*29*) force fields for protein and water molecules, respectively.

Our simulations were based on the X-ray crystal structure of AZD3152 Fab in complex with BA.2 RBD, one of the "background antigens" in our first design iteration (see Main Text). We performed mutation scan for antibody residues near the binding interface, including 19 residues on the H-chain and 12 on the L-chain. For each residue, we evaluated mutations to all alternative AA types except proline and charge-reversing substitutions. In total, these calculations covered 544 single mutations on the antibody. For each mutation, our FEP simulations predicted $\Delta\Delta G^{\text{Binding}}$ and $\Delta\Delta G^{\text{Stability}}$ (*16*) for its effects on BA.2 binding affinity and conformational stability of the antibody, respectively.

In addition, given that DMS experiments identified D420 and F456 as the main liabilities of AZD3152, we constructed eight single-point mutants of the BA.2 RBD, including four substitutions (L, N, R, Y) at D420 and four substitutions (D, L, N, V) at F456. To obtain a system of given RBD mutant in complex with AZD3152, we used a "mutating" simulation from our FEP protocol (*16*) to gradually convert the wildtype RBD residue to the new type by increasing the coupling parameter λ from 0 to 1. Then the new system was further equilibrated for 20-ns. For each RBD mutant as antigen, we performed mutation scans as described earlier, but only for antibody residues in proximity to the mutated RBD residue. Specifically, we ran FEP calculations to scan 6 and 5 antibody residues in systems with the D420 and F456 mutants, respectively, resulting in a total of 792 $\Delta\Delta G^{\text{Binding}}$ values for the various antigens.

S2.2 Methods in the first design iteration

We first provide a summary here for the first design iteration, with extensive details given in the subsections. Briefly, we first produced:

- Single and multi-point mutation antibody sequences generated by the GUIDE engine
- A set of 1,000 antibody sequences selected using a Pareto optimization-based pre-selection phase (described in Section S2.2.5.1) and evaluated with the GUIDE Engine (SFE, described in Section S2.1.2)
- 366 multi-point mutation sequences selected by combining the best performing single point mutations predicted by SFE
- 4,000 multi-point mutation sequences selected using an Integer Linear Programming optimization approach (described in Section S2.2.5.2.1).

From the above AZD3152 derivatives, we then selected 187 sequences for *in vitro* evaluation using an iterative rank-and-select approach employing a variety of utility functions (Table S7), which are defined to capture a complex set of objectives due to the large number of antigen targets and variety of affinity prediction methods used.

Additionally, we enforced "diversity constraints" such that no position is mutated in more than 25 of the 187 final selected set, and no individual mutation is present in more than 5 of the final selected set. Each step is described in detail below.

S2.2.1 Problem formulation

We follow a computational approach for antibody optimization (*11*), whereby the parental AZD3152 antibody is mutated *in silico* and assessed in simulation. The goal of the optimization is to generate a batch of diverse antibodies that achieve or maintain high affinity to the target antigen(s). Table S2 lists the mutations we introduce into BA.2 and XBB.1.5 to represent contemporary and historical variants of concern (VOCs) for which we predict $\Delta\Delta G$ values and include in the optimization. Table S6 lists the antigen variants used in $\Delta\Delta G$ calculation for each prediction tool, including natural and potential escape variants. We outline our first iteration optimization procedure in detail below.

S2.2.2 Defining the search space

By analysis of interatomic distances in the structures of AZD3152 in complex with the antigen, we selected 43 AA positions in AZD3152 for possible mutation. We allowed a position for mutation if its wildtype residue includes any atoms less than 7 Å from any antigen atom. For each position, we considered mutations to any other standard AA, barring cysteine and proline. For the given number of positions, $P = 43$, the number of allowed AAs at each, $A = 17$, and the maximum number of mutations allowed in a sequence, M, the size of the total search space is:

$$\sum_{k=1}^{M} \binom{P}{k} A^k = \sum_{k=1}^{M} \frac{P!}{k!\,(P-k)!} A^k.$$

During the first design iteration we considered up to, and including, 4 mutations ($M = 4$), resulting in a search space of 1.3×10^{10} possible mutant sequences.

S2.2.3 Defining the optimization targets

The optimization process aimed to maintain binding to wildtype BA.2, XBB.1.5, BA.2 with mutations of interest, and XBB.1.5 with mutations of interest. In total there were 11 mutations of interest for BA.2 and 11 mutations of interest for XBB.1.5 listed in Table S2, resulting in 12 antigen targets for BA.2 (including the wildtype antigen) and 12 antigen targets for XBB.1.5 (including the wildtype antigen). We also aimed to gain binding on the defined liabilities at position D420 (with mutations A, I, L, N, R) and F456 (with mutations D, L, R, N, V) with respect to BA.2 and XBB.1.5 backgrounds. In total there were 5 antigen targets for D420 per background and 5 antigen targets for F456 per background. For clarity, we separate our targets into two categories called binding targets (BT) and liability targets (LT). The respective BA.2 and XBB.1.5 antigen targets were defined as BTs, and the D420 and F456 antigen targets were defined as LTs. Our search space is defined over all possible combinations of 2, 3, and 4-point mutations starting from the parental antibody. The binding score to each LT and BT for each estimation tool is considered as an objective, resulting in 90 objectives.

S2.2.4 Evaluating antibody mutants with a surrogate function

Performing simulations described in Section S2.1 for the $\sim 10^{10}$ search space exhaustively would be prohibitive. The computational cost is further exacerbated by evaluating sequences against many antigen targets (or objectives). To overcome this limitation, we used the simulation-based approaches (Section S2.1) to individually estimate binding affinity change for each single point AA substitution at each of the selected antibody positions in complex with each of the 44 antigen targets. We then used the GUIDE engine as described in (*11*) to explore multi-point mutants. Additionally, we utilized a surrogate function, the sum-of-single-points (SSP), which linearly approximates a "score" for a given antibody mutant in complex with a given antigen target as the sum of constituent single-point substitution affinity predictions:

$$s\big((p_1, a_1, \ldots, p_k, a_k), o\big) \approx \hat{s}\big((p_1, a_1, \ldots, p_k, a_k), o\big) = \sum_{i=1}^{k} s((p_i, a_i), o),$$

where s is the score of the multi-mutation sequence, $\hat{s}$ is the sum-of-single-point score, p is a given position, a is a given AA, k is the number of mutations, and o is a given objective (antigen target). To compute any $\hat{s}(p_1, a_1, \ldots, p_k, a_k), o)$, all single point mutations from wildtype are evaluated on a given simulation tool and the corresponding $s((p_i, a_i), o)$ values tabulated for later post-hoc addition.

When $\Delta\Delta G$ is used as the score above, the SSP approximation is equivalent to the assumption of additivity of binding free energy (*16, 33*). Such approximations assume that the local environments of individual protein residues are independent of each other, thus ignoring collective or allosteric effects. Nevertheless, with the availability of single-point data, SSP can directly estimate the binding for any multi-point derivative, with negligible computational cost, thus useful for very rapid screening of large sets of antibody derivatives. Smaller sets of identified promising candidates are then re-evaluated by more accurate and computationally expensive methods.

S2.2.5 Optimization

To identify a batch of candidate mutations for *in vitro* testing that address the predefined liabilities and maintain wildtype binding, we optimized with respect to all LTs and BTs in Table S3. As aforementioned, we produced $\Delta\Delta G$ values for antibody mutants via multiple simulation methods for a portion of the search space and used the SSP surrogate for the remainder, thus leading to a "multi-fidelity" optimization scenario (*34*). Moreover, because SFE and Rosetta Flex have much

lower computational costs than FEP and PMF, they afford a much larger number of simulations allowing us to search a greater portion of the search space. To deal with multi-fidelity optimization, we used a two-fold optimization approach. First, we evaluate a large set of mutations with SSP. We then selected a fit and diverse batch of multi-mutation antibody sequences to be evaluated *in silico* in SFE simulations. This is carried out using a Pareto based multi-objective optimization approach in combination with the SSP surrogate scoring function (*35*). Second, we combined the SFE simulations with a set of proposed Rosetta Flex simulations and a further batch of sequences suggested by other optimization tools, to select a final batch of 187 fit and diverse sequences for *in vitro* testing. During each optimization stage a utility-based rank-and-select algorithm is used to select a set of candidate sequences. We outline our optimization approach in more detail below.

S2.2.5.1 Preselection

Preselection was used to propose antibody candidates for evaluation *in silico* using the SFE high fidelity simulation tool. We describe the preselection process below.

S2.2.5.1.1 Pareto front-based search

To optimize the multi-objective problem outlined in Section S2.2.3**,** we utilize Pareto front-based search. By following this approach, we consider tradeoffs for sequences that increase binding for some variants and reduce binding for others. Our multi-objective search builds an empirical 3-tier Pareto front over this search space using SSP scores (see Table S3 for every objective considered). A N-tier Pareto front can be defined as follows:

$$\bigcup_{n}^{N} PF_n$$

$$PF_n = \{ParetoFront\left(\Pi \setminus \left(\cup_{i=1}^{\max(n-1,1)}(PF_i \ \cdot \mathbf{1}_{\{n>1\}}\})\right)\right)\},$$

where $ParetoFront$ is a function to compute the Pareto front of a given set of solutions, PF is a set of Pareto efficient solutions for a given tier, and Π is the set of all possible solutions. When $\mathrm{n} = 1$, the indictor function $\mathbf{1}_{\{n>1\}}$ will return 0 and the resulting term will evaluate to an empty set ($PF_1 \cdot 0 = \emptyset$), resulting the Pareto front being computed over the full set of solutions Π (tier 1). When $\mathrm{n} > 1$ the Pareto front from all previously computed tiers is removed from the set of solutions, Π. We define $N = 3$, resulting in a 3-tier Pareto front.

Using Livermore High Performing Computing (HPC) resources, 260,967 2-point mutations, 60,631,333 3-point mutations, and 10,307,326,610 4-point mutations were computed for binding estimation. The surrogate score for each point mutation was calculated in parallel across many HPC nodes.

Although it is feasible to compute exhaustively all 2, 3, and 4-point mutations, given memory constraints it is not feasible to store such a large number of sequences in memory. By computing the 3-tier Pareto front we address the memory constraints as this computation significantly reduces the number of sequences that must be stored in memory. Table S4 presents the total number of sequences in each computed Pareto front across each tier for the specified number of mutations and objectives.

The 2, 3, and 4-point tiered Pareto fronts are then combined, and from this large number of sequences we aimed to select the 1,000 most promising ones to be evaluated with more precise

binding estimation tools. The 1,000 sequences were chosen based on utility functions in the *rank and select* operation outlined in the next subsection. The selected sequences are then evaluated using the SFE simulation tool.

S2.2.5.1.2 Rank and Select

When taking a Pareto based8 multi-objective approach to optimization, a partial ordering over solutions is computed (*36*). To further down-select those into a batch of sequences to be evaluated *in silico* using SFE, we enforce a total ordering over sequences by taking a utility-based approach (*37*). Utility functions are used to represent multiple complex objectives (*36, 37*). The utility functions take the vector of objectives (estimated binding scores) as input and return a scalar utility. Some of the utility functions aimed to capture consensus of predictions between the simulation tools, while others aim to exploit the differences in the tools representative scores. The approach was utilized to mitigate the bias and error associated with each of the simulations tools and to ensure diversity of the selected sequences. Additionally, given that it may be biophysically impossible to attain full potency to all prior, contemporary, and future SARS-CoV-2 variants in a single antibody template, our goal was to maximize variant coverage. Further, we did not have knowledge of which, if any, escape mutations might emerge in the future, thus introducing a challenge in that we did not have a clear set of optimization targets. To address this, we developed "Top N" utility functions which score an antibody sequence based on the Nth best $\Delta\Delta G$ across all of the antigen targets, thereby sacrificing the performance on some of the antigen targets in order to optimize binding to a subset of the antigen targets. This Top N approach is a risk mitigation strategy that prevents the optimization from being dominated by one or two of the most challenging antigen targets, without defining those subsets a-priori. The final batch of proposed antibodies represents multiple goals with increasing degrees of breadth.

At each iteration, the top-ranked sequence with respect to a given utility function is chosen, appended to the batch, and removed from the set of available sequences. This process repeats until a batch of sequences of fixed size is selected.

S2.2.5.1.2.1 Diversity

To enforce sequence diversity in our selected batch, we impose the following constraints:

1. Constrain the total selected sequences with a given position being mutated.
2. Constrain the total selected sequences with a given mutation.
3. Constrain the total selected sequences that contain an aromatic AA (F, Y, W).

By enforcing constraints, a diverse batch of candidates can be selected which mitigates the error and bias that is systemically present in the simulation tools. Additionally, these constraints ensure a given position or mutation is not overrepresented in the batch.

S2.2.5.1.2.2 Selection

To select 1,000 multi-point mutation sequences for evaluation *in silico* using SFE, we first remove the set of Pareto dominated sequences computed in Section S2.2.5.1.1 from the set of all possible sequences from which we select from. We then formed a set of utility functions and select sequences with respect to the defined functions. The utility functions are defined using the objectives outlined in Table S3 which capture a variety of objectives. The set of defined utility functions is outlined in Table S5. The diversity parameters for the selection process are defined as follows:

1. Maximum times a position can be selected: 200.
2. Maximum times a mutation can be selected: 40.
3. There are no constraints on the number of aromatics enforced during this selection process.

We limit our selection to a maximum of 4-point mutations from wildtype, and a mixture of 2, 3, and 4-point mutations are chosen based on utility.

S2.2.5.2 Final Selection

To select a batch of fit and diverse candidates for *in vitro* testing, our final selection process combines the multi-point designs evaluated using SFE (from Section S2.2.5.1) with exhaustive 2-point mutations evaluated using Rosetta Flex and a set of candidate sequences generated by an integer linear program. Similar to Ref. (*11*), it was feasible to exhaustively evaluate candidates with up to 2 mutations using the Rosetta Flex simulation tool. Therefore, we evaluated all possible 2-point mutations from wildtype (260,967 sequences) and utilized these high-fidelity simulations in our down-select procedure. Additionally, as mentioned above, we incorporated a batch of sequences proposed by an integer linear program algorithm. This combined set of sequences is then used by the rank-and-select algorithm to propose a final batch of 187 sequences. We describe the final selection process in more detail below.

S2.2.5.2.1 Integer linear programming

The Pareto dominance relation (described in Section S2.2.5.1) strictly removes sequences that are Pareto dominated across all Pareto tiers. While this optimization approach captures many fit candidate sequences, many may still be discarded. The simulation tools can overestimate the scores for a given mutation, and over relying on this signal during optimization can produce a batch of sequences with insufficient mutational diversity. Such an approach is risky given the fidelity gap between *in silico* simulations and *in vitro* experiments. As a result, it is important to capture as many fit and diverse mutations as possible. To do so, we explicitly make a trade-off between optimality and diversity by keeping solutions that may be Pareto dominated (suboptimal) but are diverse with respect to some characteristics of interest. To do so, we utilize integer linear programming (ILP) to generate a batch of fit and diverse sequences for down-selection. We outline the ILP formulation in the following.

Consider the matrix $A \in \mathbb{R}^{T\,x\,PA}$ of scores:

$$\mathbf{A} = \begin{pmatrix} s(1,1,1) & \dots & s(1,A,1) & s(2,1,1) & \dots & s(2,A,1) & \dots & s(P,1,1) & \dots & s(P,A,1) \\ s(1,1,2) & \dots & s(1,A,2) & s(2,1,2) & \dots & s(2,A,2) & \dots & s(P,1,2) & \dots & s(P,A,2) \\ \vdots & \ddots & \vdots & \vdots & \ddots & \vdots & \vdots & \ddots & \vdots & \vdots \\ s(1,1,T) & \dots & s(1,A,T) & s(2,1,T) & \dots & s(2,A,T) & \dots & s(P,1,T) & \dots & s(P,A,T) \end{pmatrix}$$

and the vector $X \in \mathbb{R}^{PA\,x\,1}$ of variables:

$$\mathbf{X} = \begin{pmatrix} x(1,1) & \dots & x(1,A) & x(2,1) & \dots & x(2,A) & \dots & x(P,1) & \dots & x(P,A) \end{pmatrix}$$

where $x(p,\, a) \in \{0,\, 1\}$ is the variable corresponding to the mutation at position p to amino acid a.

The problem can then be defined as follows:

$$\begin{aligned} \text{Maximize} \quad & \mathbf{A} \cdot \mathbf{X}^T \\ \text{subject to} \quad & 0 \le \sum_{a=1}^{A} x(p,a) \le 1 \quad \forall p \in \{1,\ldots,P\} \\ & m \le \mathbf{1}\mathbf{X}^T \le M \\ & \mathbf{X} \in \{0,1\}^{PA} \end{aligned}$$

where:

- $A \cdot X$ is a vector of size O
- $0 \le \sum_{a=1}^{A} x(p,a) \le 1 \; \forall p \in \{1, \ldots, P\}$ where P is the set of constraints
- $m \le 1\, X^T \le M$ is a constraint enforcing the number of mutations is between m and M.

The problem definition above returns a vector of size O. To select sequences a total ordering over solutions must be computed, therefore we must scalarize the problem using a weight vector w, where a given weight over an objective represents the preference. This reduces the multi-objective problem to a single-objective problem which the ILP can optimally solve.

We define w as follows:

$$w = (w_1, \ \ldots, w_O),$$

where w is a vector of linear weights (convex combination) with a weight per objective o. Therefore, the optimization problem can be formulated and solved as follows:

$$\begin{aligned} \text{Maximize} \quad & (\mathbf{w} \cdot \mathbf{A}) \cdot \mathbf{X}^T \\ \text{subject to} \quad & 0 \le \sum_{a=1}^{A} x(p,a) \le 1 \quad \forall p \in \{1,\ldots,P\} \\ & m \le \mathbf{1}\mathbf{X}^T \le M \\ & \mathbf{X} \in \{0,1\}^{PA} \end{aligned}$$

This problem formulation can be used to iteratively select optimal solutions with respect to the objectives and scoring function. The ILP is utilized to generate a batch of K sequences with respect to the constraints and weight vector w. To ensure exploration of the search space, the weight vector w is randomly selected at each iteration. At each iteration of the ILP optimizes with respect to different preferences over the problem objectives, thereby ensuring exploration.

We use the ILP to generate a batch of 4,100 3-point mutations from wildtype (AZD3152) which are used in the rank and select procedure in Section S2.2.5.2.2. The ILP optimizes for all objectives listed in Table S6 (except for Rosetta Flex and PMF objectives), which totals 98 objectives. Furthermore, the ILP formulation uses the following constraints to ensure diversity:

1. Maximum times a position can be selected: 300.
2. Maximum times a mutation can be selected: 200.

The sequences generated by the ILP are then used in the rank and select procedure in Section S2.2.5.2.2, where a final batch of sequences are selected for *in vitro* testing.

S2.2.5.2.2 Rank and Select

Similar to Section S2.2.5.1.2, we use a rank and select algorithm to select a batch of 187 sequences for *in vitro* testing. Furthermore, we also include diversity constraints when selecting the final batch of 187 sequences. We define our approach in more detail below.

S2.2.5.2.2.1 Diversity

To diversify our batch, we enforce the same constraints as defined in Section S2.2.5.1.2.1.

S2.2.5.2.2.2 Selection

To select sequences for evaluation *in vitro* we first combine the set of 1,000 antibody sequences generated by the preselection phase (described in Section S2.2.5.1), the set of sequences generated by the GUIDE Engine with Rosetta Flex, the 366 multi-point mutation sequences selected by SFE (described in Section S2.1.2), and 4,000 multi-point mutation sequences selected using the ILP optimization tool (described in Section S2.2.5.2.1).

We use a set of utility functions to select sequences with respect to the defined functions. The utility functions are defined using the objectives presented in Table S6. The set of defined utility functions is outlined in Table S7.

The diversity constraints for the selection process are parameterized as follows:

1. Maximum times a position can be selected: 25.
2. Maximum times a mutation can be selected: 5.
3. Maximum allowed aromatics: 90.

Z-scores, with respect to each simulation tool, are computed and utilized in the utility functions. The Z-score, Z(s), for a given sequence, s, with respect to a given tool, t, can be computed as follows:

$$Z(s) = \frac{s - \bar{t}}{\sigma_t}$$

where $\bar{t}$ is the mean ddG value for a given tool, t, and σ_t is the ddG standard deviation for tool t.

We limit our selection to a maximum of 3-point mutations from wildtype. We remove sequences from consideration that contain more than two newly introduced aromatic residues. Our final selection contained 66 1-point mutations, 80 2-point mutations, and 40 3-point mutations.

S2.3 Methods in the second design iteration

In the second iteration, we selected and combined favorable mutations experimentally identified in the first iteration via direct analysis of binding affinity values and computational methods. We further enriched this set by including an explicitly optimized selection of mutations designed to enhance sequence positional diversity. We refer to the procedure performed in the second iteration as "*select, combine and diversify*" strategy. To account for any batch effects between first- and second-iteration experiments, we also introduced three longitudinal bridging sequences into the

final set. The goal in this iteration was to produce a list of 188 antibody mutants that would include one or more designs that increase binding affinity to the AZD3152 escape variants, prioritizing the XBB.1.5+456L target, to a level comparable to the affinity of the AZD3152 base antibody to the XBB.1.5 antigen. Concretely, this level was estimated to be an approximately 40-50-fold increase in binding signal over the AZD3152 antibody to XBB.1.5+456L.

The selection phase involves identifying and choosing the most effective single and double-point mutations that resulted in the greatest improvement in binding affinity to the target. This selection process is based on a comprehensive analysis of both experimental data from first iteration and computational insights.

In the subsequent combination phase, we conducted an exhaustive amalgamation of the mutation sets identified during the selection phase. This process included scrutiny for antibody mutations occurring at the same sequence position, removal of any duplicate sequences, avoidance of combinations of mutations that interact with the same antigen AA (positional clash) and non-bridging sequences evaluated in the first iteration.

S2.3.1 Select

S2.3.1.1 Selection based on directly measured binding affinity

Following the assessment of single and double-point mutants in the experimental data from "Iteration 1", we categorized mutations into two tiers based on their binding affinity to the target XBB.1.5+456L. In Tier 1 (T1), we incorporated mutations that exhibited a substantial enhancement, at least tenfold, in binding affinity compared to the reference parental antibody. Tier 2 (T2) comprised mutations with binding affinity improvements relative to the reference antibody, although falling below the tenfold threshold. Three antibody mutants were selected to be part of Tier 1 (2 single-point and 1 double-point mutants), while Tier 2 contained 9 mutants (8 single-point and 1 triple-point mutants).

S2.3.1.2 Selection based on inferred binding affinity

Since not all individual mutations were assessed in the first iteration as antibodies with just this single mutation differentiating them from the parental AZD3152, we employed a computational approach to infer potentially beneficial single-point mutations by drawing insights from the evaluation of multi-point mutations in the same iteration. For instance, if a double-point mutation resulted in weak binding, but the introduction of an additional mutation led to an increased binding affinity for the triple-point mutant, the latter mutation would emerge as a promising Tier 2 mutation candidate. To implement this strategy, we designed a sparse regression analysis to computationally identify promising single-point mutations. In this methodology, mutants were represented as one-hot-encoded vectors, each with a dimensionality (D) corresponding to the size of the dictionary of all available single-point and double-point mutations within the "Iteration 1" dataset. Each antibody mutant was characterized as a D-dimensional binary vector, with 0's in all positions except for those where the specific mutation was present within the mutant. The dependent variable was the measured binding affinity for the target antigen. Subsequently, we trained a LASSO model (*38*) on this encoded data, identifying mutations with associated positive, non-zero weights in the model as promising candidates. This process revealed 14 promising mutation candidates; 10 of these candidates were already included in Tier 1 and Tier 2, resulting in the generation of 4 additional single-point mutations. As inputs to the *Combine* phase, we treated the computationally identified mutations to comparable to those in the Tier 2 set identified directly from the empirical

data. This combined empirical and inferred set is henceforth referred to as the "Extended Tier 2" (ET2) set.

S2.3.1.3 Selection based on expert investigation

In addition to the T1 and ET2 sets, structural biologists identified a third set with mutations deemed potentially advantageous. Expert analysis suggested two specific single-point mutations as possible major contributors against the related target XBB.1.5+420L, aiming to bolster binding affinity to XBB.1.5+456L. This set of two mutations is denoted as XS (expert-selected). Experimental results later revealed that these mutations are not featured in the top performing candidates.

S2.3.2 Combine

The sets of favorable mutations identified in the *Select* phase serve as the building blocks for creating multi-point mutants that will together constitute the final set of antibody mutants. In this "Combine" phase, we generated a diverse and comprehensive set of mutants. This step is essential to ensure that we cover a broad selection of potential mutations with the aim of improving binding affinity.

Several combination strategies were executed, each designed to leverage different aspects of the selected mutations. Table S8 shows the list of strategies employed. The underlying logic guiding these combination strategies is to produce antibody mutants with increased binding affinities through the combination of either robust binders with moderate ones or multiple moderate binders.

The sets were produced following the strategy order outlined in Table S8. To ensure uniqueness, mutants resulting from each strategy were checked against the already generated mutants by previous strategies and duplicates were removed. Strategy 2 and 3 differ by the fact that Strategy 2 mitigated the risk of previously untested combinations of mutations by disallowing positional clashes (two or more antibody mutations in close proximity with the same antigen AA). In contrast, Strategy 3 relaxed this constraint, accommodating mutants that may involve clashes within antibody regions of interest. All other strategies remained consistent in not permitting positional clashes.

Aside from the mutants generated by the combination procedure described above, a small set of antibody mutants were proposed based on our intuition for structural biology, by combining XS mutations and some of the mutations in the Tier 1 set. This process generated additional 23 mutants: 4 single-point mutants, 3 double-point mutants, 8 three-point mutants and 8 four-point mutants. In subsequent experimental validation, none of these mutants resulted in sufficiently strong binding to be included in the top candidates.

As previously noted, three single-point mutants tested in the first iteration were also included in the second iteration. This allows for bridging between these sets of data and would have supported quantitative calibration in the event a third iteration was required.

S2.3.3 Diversify

To further enhance the diversity within the final set of mutants, we introduced a diversity-driven optimization-based approach to curate the remaining mutants that would constitute our final set. This process involved several key steps: First, we initiated the process by exhaustively generating all possible quadruple-point combinations of mutations found within the ET2 set. Next, we pruned this extensive set of combinations by 1) removing duplicates from previous steps, 2) removing

mutants bearing multiple mutations at the same sequence position, 3) deleting mutants that had already been evaluated in the first iteration 1, and 4) removing those mutants with positional clashes. 281 unique antibody mutants remained at this stage.

We used a Genetic Algorithm (GA) to strategically choose a subset of 41 quadruple-point mutants, from this set of 281, that provided the maximum coverage of positional mutations, effectively addressing the “n choose k” problem, with n=281 and k=41. The number 41 was determined by subtracting the number of already generated mutants by the combination, expert selection, and bridging (147) from total number of antibody mutants to be generated in this iteration (188). The GA fitness function was defined as the inverse of the variance of mutation counts for each mutable position within the antibody sequence. The GA was executed with a population of 100 individuals and ran for 500 generations. This resulted in a subset of 41 quadruple-point mutants that maximized diversity.

The final set of antibody mutants from the Iteration 2 was assembled by concatenating the four sets described above: 1) mutants resulting from the “Combine” phase (121); 2) expert-selected mutants (23); 3) diversity-optimized mutants (41); and 4) bridging mutants (3). Figure S1 presents the distribution of the number of mutations in the mutants generated in the iteration 2.

After the completion of the project, as a retrospective assessment of our approach, we examined the top candidates selected by the subsequent experimental screening, revisiting how they were generated in this computational design iteration. Among the four sets of generated antibody mutants described earlier, all our top 20 candidates (Table 1) belong to the first set (i.e., by combining favorable mutations) and none came from the other three. More specifically, as explained in Section S2.3.2, we employed seven strategies (Table S8) to generate the 121 antibody mutants in that set. Strategies 1, 2, 3, and 7 contributed 2, 5, 12, and 1 candidates in the top-20 list, respectively. In particular, two of our top four candidates (Table 3) originated from strategy 2 and the other two (including 3152-1142) from strategy 3.

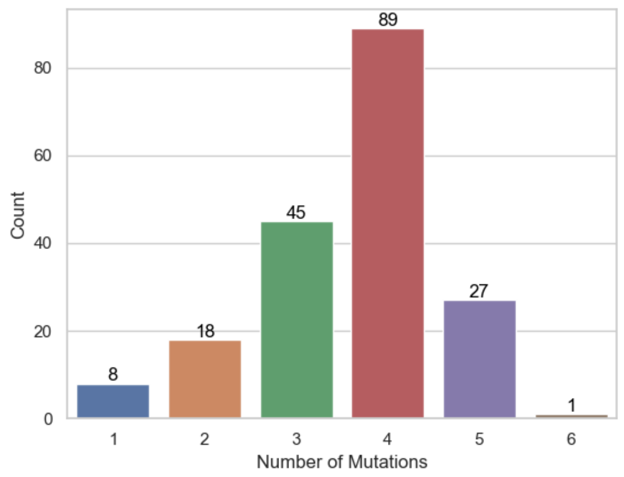

Figure S1. Distribution of the number of mutations in the final set of 188 antibody mutants generated in the second iteration.

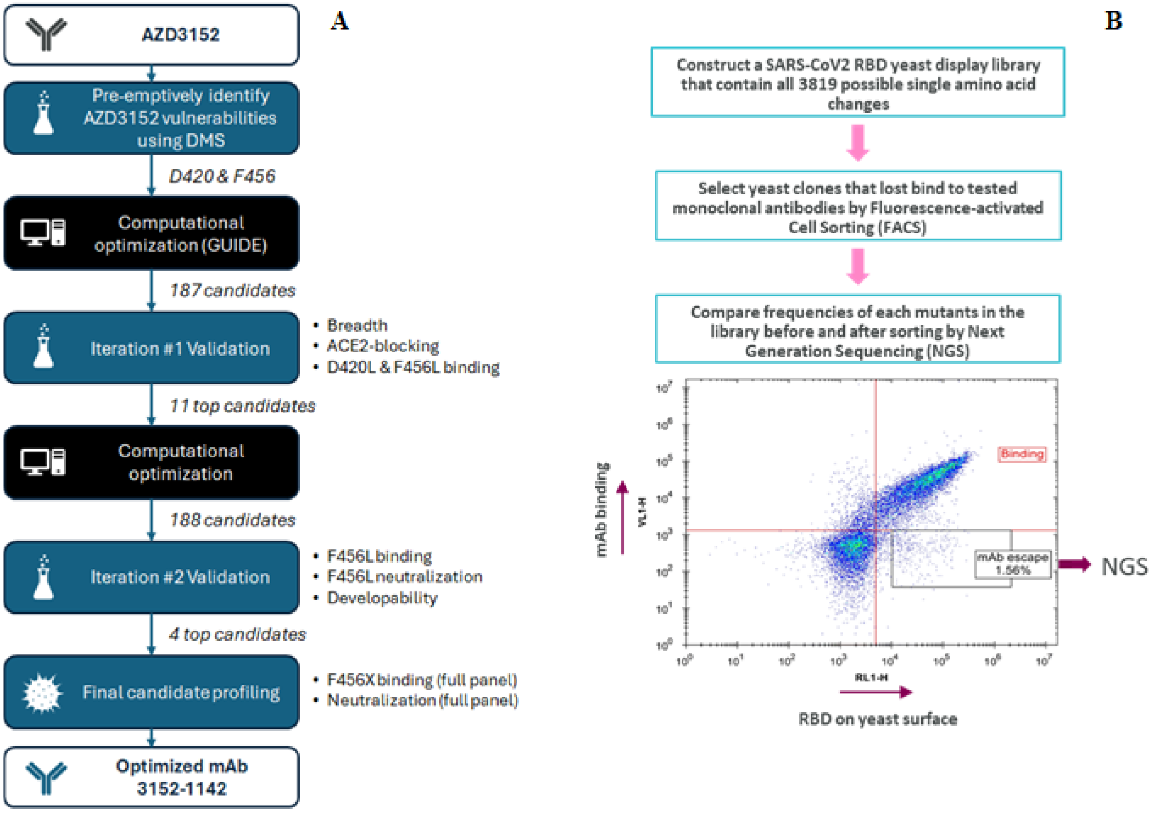

Figure S2. (**A**) Overall workflow in this study. (**B**) Schematic for deep mutational scanning experiments.

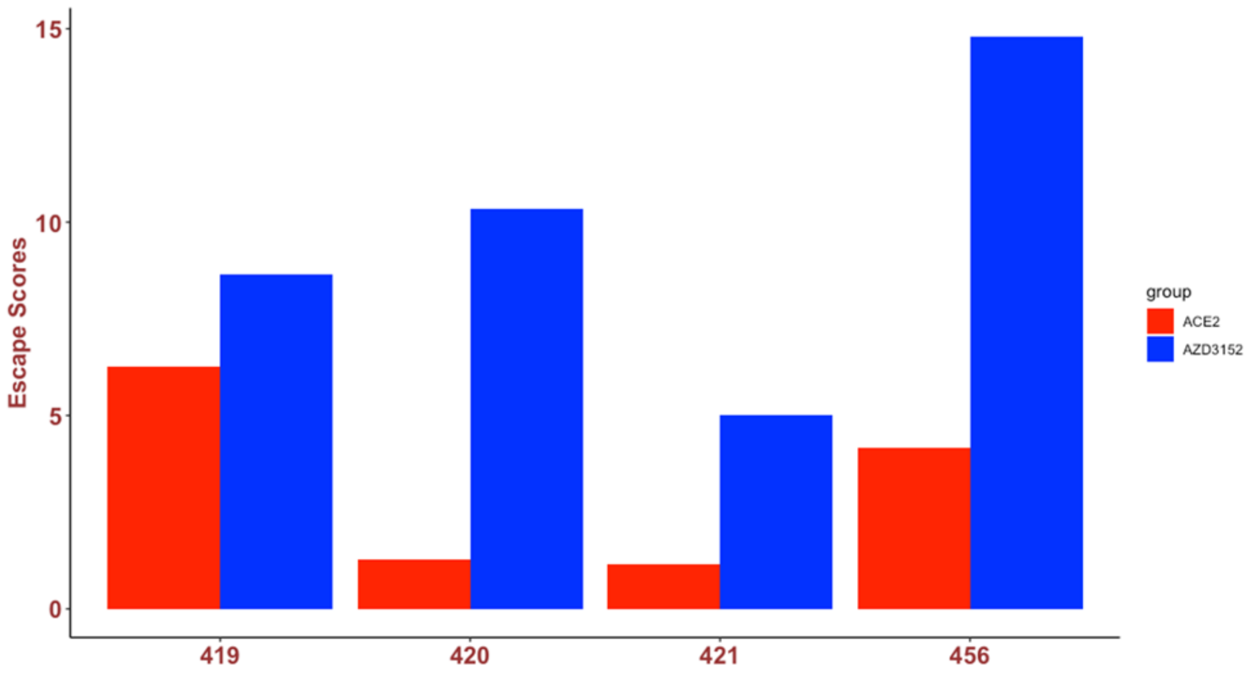

Figure S3. Summed escape scores for four residues comparing AZD3152 and ACE2. Escape scores for all mutations at down-selected residues critical for AZD3152 (*blue*) binding were summed and compared with summed escape scores for ACE2 (*red*).

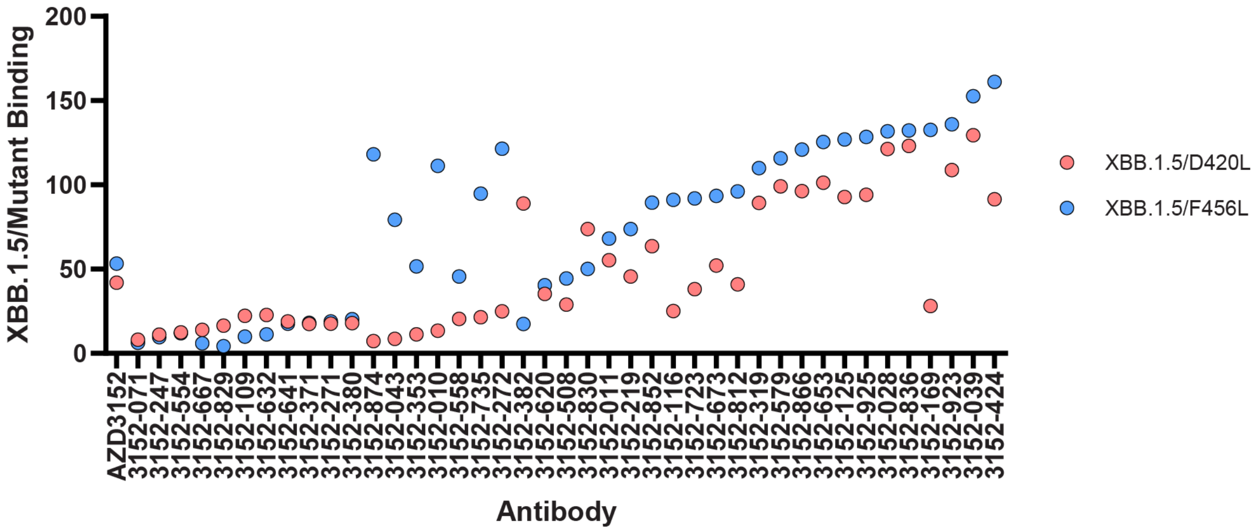

Figure S4. FACS-based binding fold changes for 41 designed derivatives in the first design iteration. The fold changes are for XBB.1.5+D420L or XBB.1.5+F456L compared to the binding between parental AZD3152 and wildtype XBB.1.5. The same graph for the 11 best derivatives is shown in Fig. 2D.

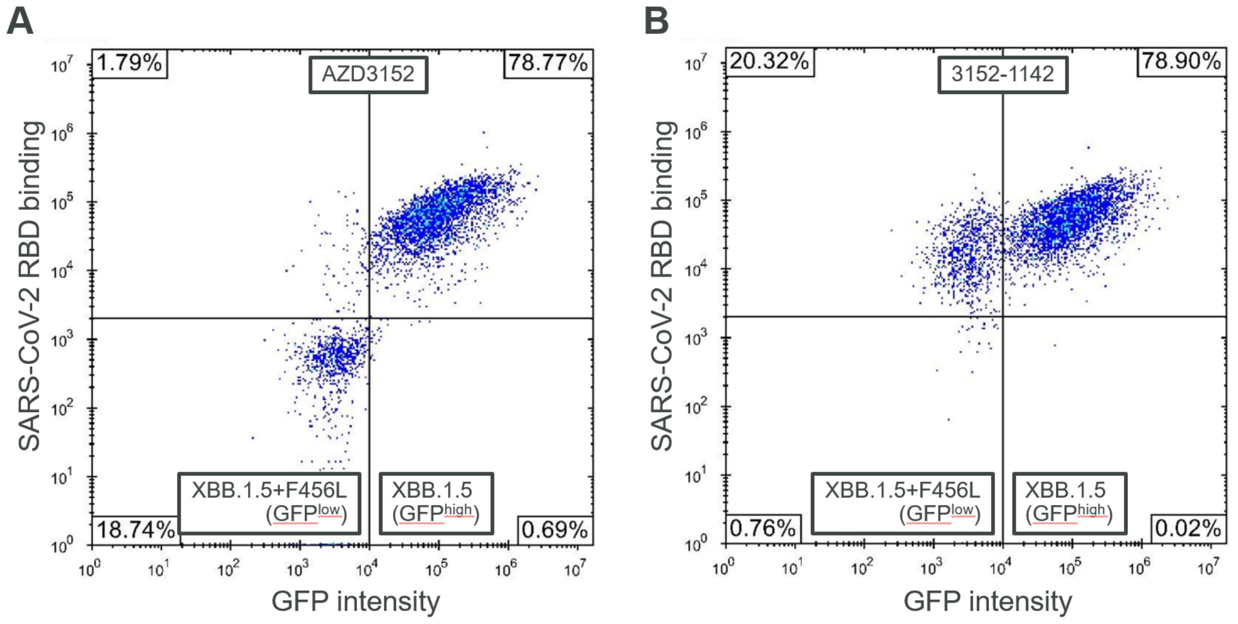

Figure S5. Yeast co-culture assay comparing the binding of AZD3152 (**A**) and a sample derivative mAb (**B**) to XBB.1.5 RBD and XBB.1.5+F456L RBD. Yeast constitutively expressing GFP and displaying XBB.1.5 RBD is mixed with non-fluorescent yeast displaying XBB.1.5+F456L. Staining of the mixture with mAb (0.1µg/mL) is detected via fluorescent anti-Fc secondary antibody. Improvements in binding are quantified by comparing the geometric means of the GFP^{low} and GFP^{high} populations.

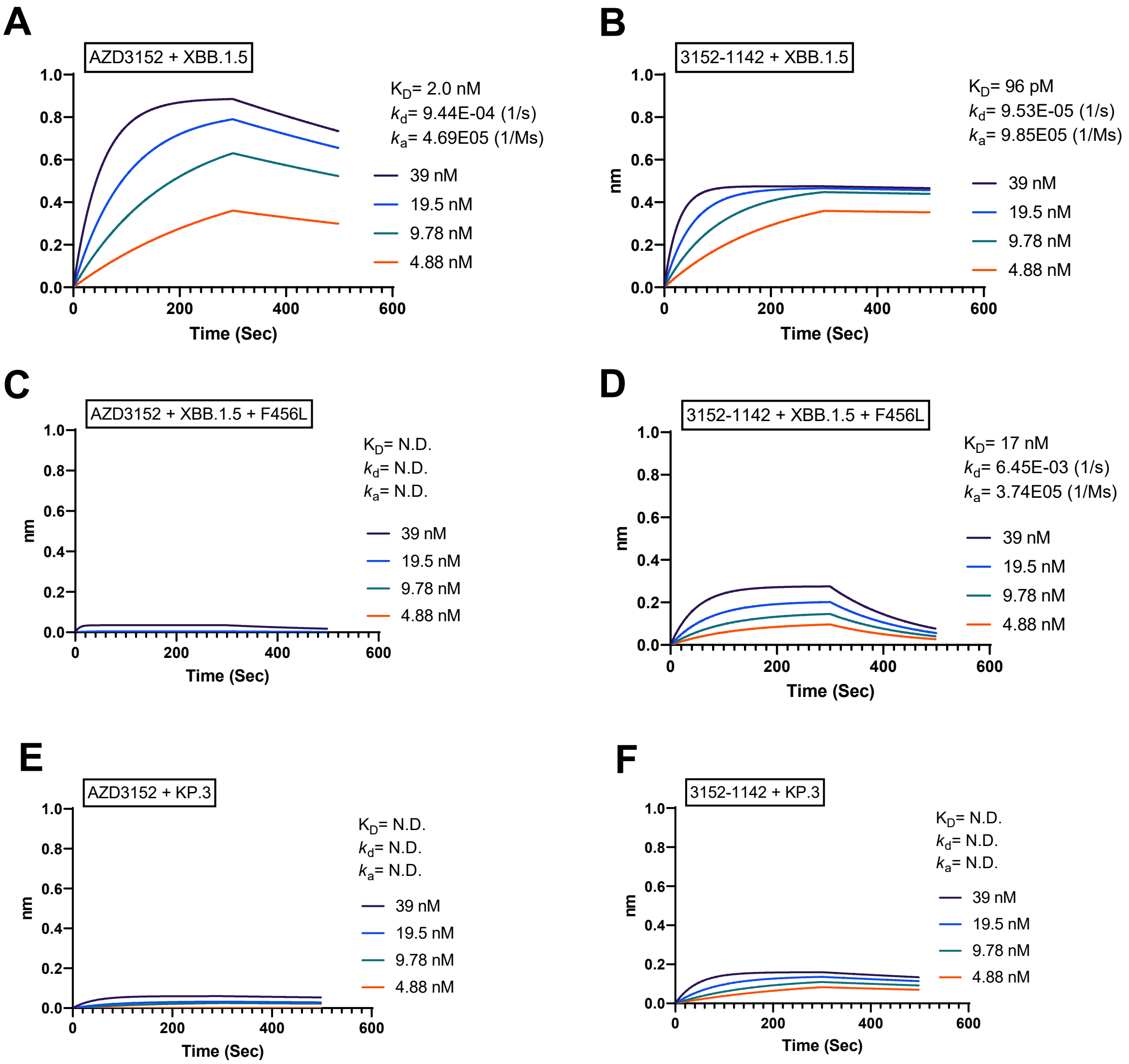

Figure S6. Binding kinetics of AZD3152 and 3152-1142 to SARS-CoV-2 RBDs. Kinetic parameters were measured by biolayer interferometry (BLI). (**A**, **C**, **E**) BLI kinetic measurements using AZD3152 loaded onto Octet probes and associated with varying concentrations of XBB.1.5 RBD (A), XBB.1.5+F456L RBD (C), and KP.3 RBD (E). The same RBDs were also tested for binding to 3152-1142 (**B**, **D**, **F**). Fits were determined using a 1:1 binding model for response signals over 0.20 nm; others are listed as "N.D." (not determined).

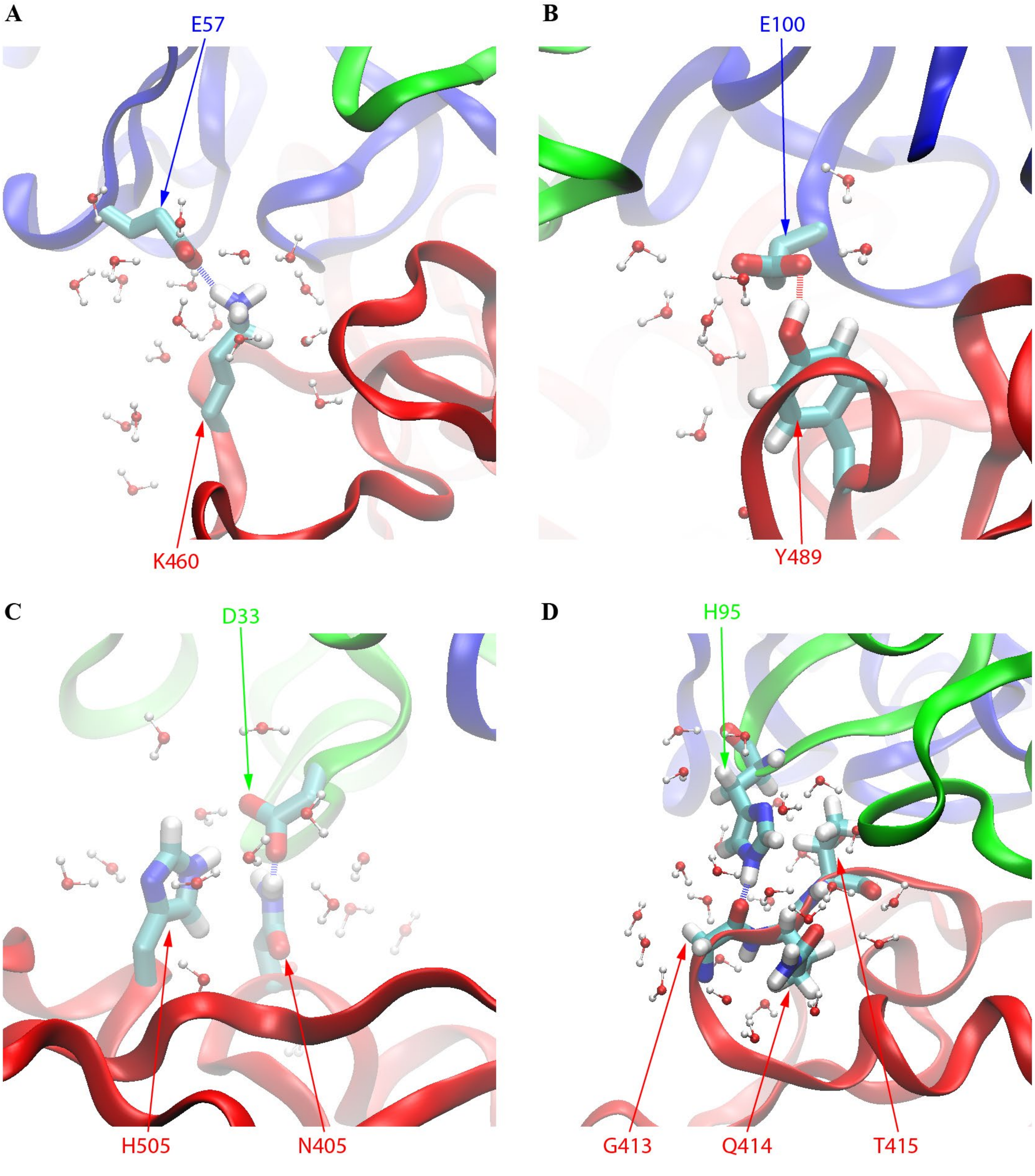

Figure S7. MD snapshots highlighting interactions involving some of the mutated residues in 3152-1142. The snapshots were taken from an equilibrium MD simulation of the XBB.1.5 RBD and the 3152-1142 Fab. The backbones of the RBD and the HC and LC of the Fab are displayed in *red*, *blue*, and *green*, respectively. Compared to the parental AZD3152, the following substituted AAs are shown: (**A**) HC_S57E; (**B**) HC_A100E; (**C**) LC_N33D; (**D**) LC_G95H.

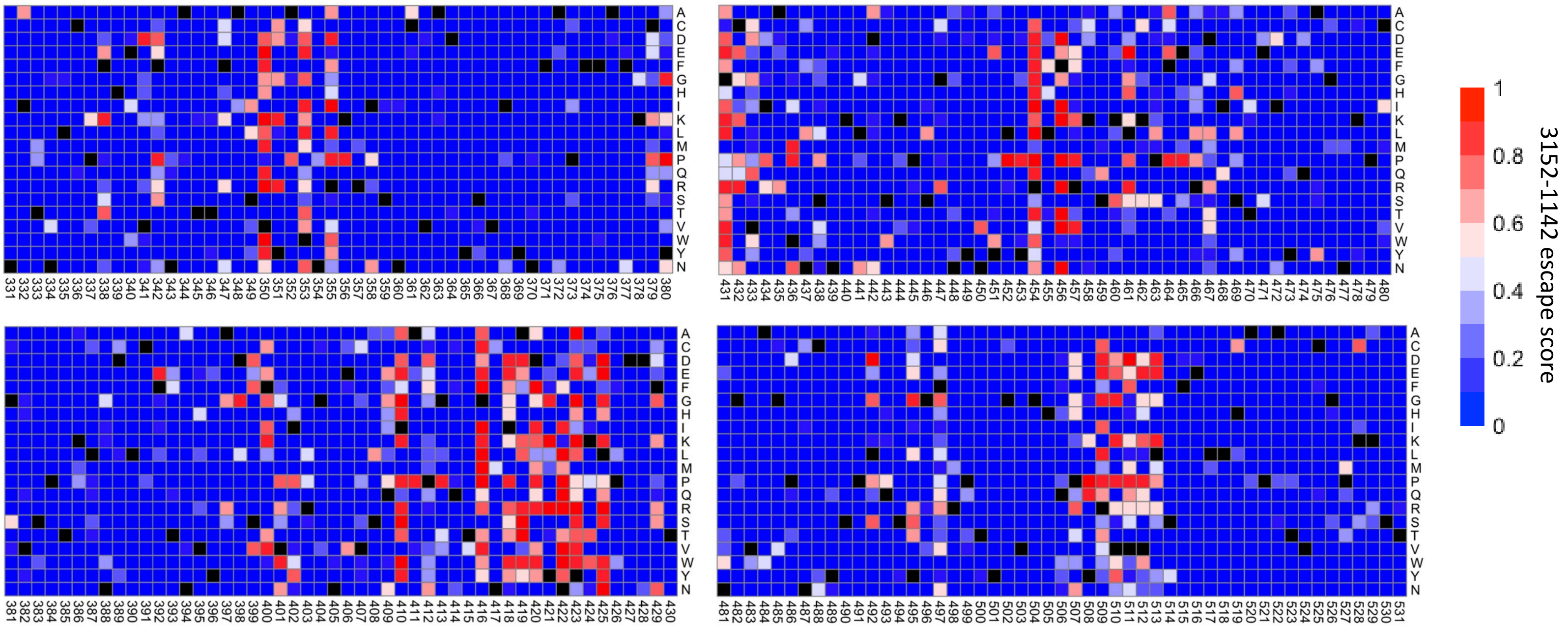

Figure S8. Heatmap illustrating the impact of every mutation on 3152-1142 binding. Black squares represent the wildtype AA for a particular residue.

Table S1. SARS-CoV-2 variant spike amino acid mutations for pseudovirus neutralization assay. All SARS-CoV-2 variant pseudoviruses were produced using a lentiviral-based system. The spike amino acid mutations that are different from the ancestral Wuhan strain are listed.

Variant	Spike mutations
D614G	D614G
Alpha	del69 -70, del144, N501Y, A570D, D614G, P681H, T716I, S982A, D1118H
Beta	L18F, D80A, D215G, del241-243, K417N, E484K, N501Y, D614G, A701V
Delta	T19R, T95I, G142D, del156-157, R158G, L452R, T478K, D614G, P681R, D950N
Gamma	L18F, T20N, P26S, D138Y, R190S, K417T, E484K, N501Y, D614G, H655Y, T1027I, V1176F
BA.1	A67V, del69-70, T95I, G142D, del143-145, del211, L212I, ins214EPE, G339D, S371L, S373P, S375F, K417N, N440K, G446S, S477N, T478K, E484A, Q493R, G496S, Q498R, N501Y, Y505H, T547K, D614G, H655Y, N679K, P681H, N764K, D796Y, N856K, Q954H, N969K, L981F
BA.1.1	A67V, del69-70, T95I, G142D, del143-145, del211, L212I, ins214EPE, G339D, R346K, S371L, S373P, S375F, K417N, N440K, G446S, S477N, T478K, E484A, Q493R, G496S, Q498R, N501Y, Y505H, T547K, D614G, H655Y, N679K, P681H, N764K, D796Y, N856K, Q954H, N969K, L981F
BA.2	T19I, del24-26, A27S, G142D, V213G, G339D, S371F, S373P, S375F, T376A, D405N, R408S, K417N, N440K, S477N, T478K, E484A, Q493R, Q498R, N501Y, Y505H, D614G, H655Y, N679K, P681H, N764K, D796Y, Q954H, N969K
BA.2.12.1	T19I, del24-26, A27S, G142D, V213G, G339D, S371F, S373P, S375F, T376A, D405N, R408S, K417N, N440K, L452Q, S477N, T478K, E484A, Q493R, Q498R, N501Y, Y505H, D614G, H655Y, N679K, P681H, S704L, N764K, D796Y, Q954H, N969K
BA.4/5	T19I, del24-26, A27S, del69-70, G142D, V213G, G339D, S371F, S373P, S375F, T376A, D405N, R408S, K417N, N440K, L452R, S477N, T478K, E484A, F486V, Q498R, N501Y, Y505H, D614G, H655Y, N679K, P681H, N764K, D796Y, Q954H, N969K
BA.2.75	T19I, del24-26, A27S, G142D, K147E, W152R, F157L, I210V, V213G, G257S, G339H, S371F, S373P, S375F, T376A, D405N, R408S, K417N, N440K, G446S, N460K, S477N, T478K, E484A, Q498R, N501Y, Y505H, D614G, H655Y, N679K, P681H, N764K, D796Y, Q954H, N969K
BQ.1.1	T19I, del24-26, A27S, del69-70, G142D, V213G, G339D, R346T, S371F, S373P, S375F, T376A, D405N, R408S, K417N, N440K, K444T, L452R, N460K, S477N, T478K, E484A, F486V, Q498R, N501Y, Y505H, D614G, H655Y, N679K, P681H, N764K, D796Y, Q954H, N969K
XBB.1	T19I, del24-26, A27S, V83A, G142D, del144, H146Q, Q183E, V213E, G252V, G339H, R346T, L368I, S371F, S373P, S375F, T376A, D405N, R408S, K417N, N440K, V445P, G446S, N460K, S477N, T478K, E484A, F486S, F490S, Q498R, N501Y, Y505H, D614G, H655Y, N679K, P681H, N764K, D796Y, Q954H, N969K
XBB.1.5	T19I, del24-26, A27S, V83A, G142D, delY144, H146Q, Q183E, V213E, G252V, G339H, R346T, L368I, S371F, S373P, S375F, T376A, D405N, R408S, K417N, N440K, V445P, G446S, N460K, S477N, T478K, E484A, F486P, F490S, Q498R, N501Y, Y505H, D614G, H655Y, N679K, P681H, N764K, D796Y, Q954H, N969K

XBB.1.16	T19I, del24-26, A27S, V83A, G142D, del144, H146Q, E180V, Q183E, V213E, G252V, G339H, R346T, L368I, S371F, S373P, S375F, T376A, D405N, R408S, K417N, N440K, V445P, G446S, N460K, S477N, T478R, E484A, F486P, F490S, Q498R, N501Y, Y505H, D614G, H655Y, N679K, P681H, N764K, D796Y, Q954H, N969K
XBB.1.5.10	T19I, del24-26, A27S, V83A, G142D, del144, H146Q, Q183E, V213E, G252V, G339H, R346T, L368I, S371F, S373P, S375F, T376A, D405N, R408S, K417N, N440K, V445P, G446S, F456L, N460K, S477N, T478K, E484A, F486P, F490S, Q498R, N501Y, Y505H, D614G, H655Y, N679K, P681H, N764K, D796Y, Q954H, N969K
EG.5.1	T19I, del24-26, A27S, Q52H, V83A, G142D, del144, H146Q, Q183E, V213E, G252V, G339H, R346T, L368I, S371F, S373P, S375F, T376A, D405N, R408S, K417N, N440K, V445P, G446S, F456L, N460K, S477N, T478K, E484A, F486P, F490S, Q498R, N501Y, Y505H, D614G, H655Y, N679K, P681H, N764K, D796Y, Q954H, N969K
BA.2.86	Ins16MPLF, T19I, R21T, del 24-26, A27S, S50L, del 69-70, V127F, G142D, del144, F157S, R158G, del211, L212I, V213G, L216F, H245N, A264D, I332V, G339H, K356T, S371F, S373P, S375F, T376A, R403K, D405N, R408S, K417N, N440K, V445H, G446S, N450D, L452W, N460K, S477N, T478K, N481K, del483, E484K, F486P, Q498R, N501Y, Y505H, E554K, A570V, D614G, P621S, H655Y, N679K, P681R, N764K, D796Y, S939F, Q954H, N969K, P1143L
JN.1	ins16MPLF, T19I, R21T, del 24-26, A27S, S50L, H69del, V70del, V127F, G142D, Y144del, F157S, R158G, N211del, L212I, V213G, L216F, H245N, A264D, I332V, G339H, K356T, S371F, S373P, S375F, T376A, R403K, D405N, R408S, K417N, N440K, V445H, G446S, N450D, L452W, L455S, N460K, S477N, T478K, N481K, V483del, E484K, F486P, Q498R, N501Y, Y505H, E554K, A570V, D614G, P621S, H655Y, N679K, P681R, N764K, D796Y, S939F, Q954H, N969K, P1143L
JN.1.16	ins16MPLF, T19I, R21T, del 24-26, A27S, S50L, H69del, V70del, V127F, G142D, Y144del, F157S, R158G, N211del, L212I, V213G, L216F, H245N, A264D, I332V, G339H, K356T, S371F, S373P, S375F, T376A, R403K, D405N, R408S, K417N, N440K, V445H, G446S, N450D, L452W, L455S, F456L, N460K, S477N, T478K, N481K, V483del, E484K, F486P, Q498R, N501Y, Y505H, E554K, A570V, D614G, P621S, H655Y, N679K, P681R, N764K, D796Y, S939F, Q954H, N969K, P1143L
KP.2	ins16MPLF, T19I, R21T, del 24-26, A27S, S50L, H69del, V70del, V127F, G142D, Y144del, F157S, R158G, N211del, L212I, V213G, L216F, H245N, A264D, I332V, G339H, R346T, K356T, S371F, S373P, S375F, T376A, R403K, D405N, R408S, K417N, N440K, V445H, G446S, N450D, L452W, L455S, F456L, N460K, S477N, T478K, N481K, V483del, E484K, F486P, Q498R, N501Y, Y505H, E554K, A570V, D614G, P621S, H655Y, N679K, P681R, N764K, D796Y, S939F, Q954H, N969K, V1104L, P1143L
KP.3	ins16MPLF, T19I, R21T, del 24-26, A27S, S50L, H69del, V70del, V127F, G142D, Y144del, F157S, R158G, N211del, L212I, V213G, L216F, H245N, A264D, I332V, G339H, K356T, S371F, S373P, S375F, T376A, R403K, D405N, R408S, K417N, N440K, V445H, G446S, N450D, L452W, L455S, F456L, N460K, S477N, T478K, N481K, V483del, E484K, F486P, Q493E, Q498R, N501Y, Y505H, E554K, A570V, D614G, P621S, H655Y, N679K, P681R, N764K, D796Y, S939F, Q954H, N969K, V1104L, P1143L

Table S2. Mutations of interest introduced into BA.2 and XBB.1.5 backgrounds in the first computational design iteration (Section S2.2) to represent contemporary and historical SARS-CoV-2 variants.

Wildtype	RBD mutations of interest
BA.2	N405D
	S408R
	N417K
	N417T
	N460K
	N477S
	F486P
	F486S
	F486V
	R493K
	R493Q
XBB.1.5	N405D
	S408R
	N417K
	N417T
	K460N
	N477S
	S486F
	S486P
	S486V
	Q493K
	Q493R

Table S3. All objectives (90) used for the Pareto front optimization outlined in Section S2.2.5.1.

Simulation Tool	Background	Structure	BT	LT	Total BT Objectives	Total LT Objectives	Total Objectives
SFE	BA.2	7xb0	Wildtype, F486P, F486S, F486V, N405D, N417K, N417T, N460K, N477S, R493K, R493Q, S408R	D420A, D420I, D420L, D420N D420R, F456D, F456L, F456N, F456R, F456V	12	10	22
SFE	BA.2	CryoEM	Wildtype, F486P, F486S, F486V, N405D, N417K, N417T, N460K, N477S, R493K, R493Q, S408R	D420A, D420I, D420L, D420N D420R, F456D, F456L, F456N, F456R, F456V	12	10	22
SFE	XBB.1.5	7xb0	Wildtype, K460N, N405D, N417K, N417T, N477S, Q493K, Q493R, S408R, S486F, S486P, S486V	D420A, D420I, D420L, D420N D420R, F456D, F456L, F456N, F456R, F456V	12	10	22
SFE	XBB.1.5	CryoEM	Wildtype, K460N, N405D, N417K, N417T, N477S, Q493K, Q493R, S408R, S486F, S486P, S486V	D420A, D420I, D420L, D420N D420R, F456D, F456L, F456N, F456R, F456V	12	10	22
FEP Stability	BA.2	N/A	Wildtype	N/A	1	0	1
FEP	BA.2	N/A	Wildtype	N/A	1	0	1
				Total	50	40	90

Table S4. The total number of sequences contained within each tiered Pareto front for a given number of mutations and objectives.

Number of mutations	Pareto tier	Number of objectives	Total Number of Sequences
2	1	90	1,734
	2		5,306
	3		9,150
3	1	90	13,679
	2		53,267
	3		107,206
4	1	30	6,042
	2		19,006
	3		36,470

Table S5. Utility functions used to select 1,000 antibody sequences for multipoint simulations in Section S2.2.5.1. Max utility function scores an antibody based on its worst $\Delta\Delta G$ across the antigen targets, which drives optimization to maximize binding to all antigen targets (i.e., a conservative $\Delta\Delta G$ estimate). Min utility function scores an antibody based on the best $\Delta\Delta G$ across the antigen targets, maximizing binding to just a single antigen target. Mean top N utility functions score an antibody sequence by taking the N best $\Delta\Delta G$ across all of the antigen targets and computing the mean over the antigen targets, thereby sacrificing the performance on some of the antigen targets in order to optimize binding to a subset of the antigen targets. This mean top N approach is a risk mitigation strategy that prevents the optimization from being dominated by the most challenging antigen targets, without defining those subsets a-priori.

**Utility Function**	**Description**
Max	Score antibody using worst case binding across all antigen targets
Min	Score antibody using best case binding across all antigen targets
Avg	Score antibody using average binding across all antigen targets
Mean top N for N = 3, 5, 7	Score antibody based on the mean Nth best ddG across all antigen targets,
Mean top N (at antibody positions not interacting with D420 and F456), for N = 3, 5, 7	Score antibody based on the mean Nth best ddG across all antigen targets, only mutating antibody positions not interacting with D420 & F456

Table S6. All computed objectives used for 187 sequence selection outlined in Section S2.2.5.2.

Simulation Tool	Background	Structure	BT	LT	Total BT Objectives	Total LT Objectives	Total Objectives
SFE	BA.2	7xb0	Wildtype, F486P, F486S, F486V, N405D, N417K, N417T, N460K, N477S, R493K, R493Q, S408R	D420A, D420I, D420L, D420N D420R, F456D, F456L, F456N, F456R, F456V	12	10	22
SFE	BA.2	CryoEM	Wildtype, F486P, F486S, F486V, N405D, N417K, N417T, N460K, N477S, R493K, R493Q, S408R	D420A, D420I, D420L, D420N D420R, F456D, F456L, F456N, F456R, F456V	12	10	22
SFE	XBB.1.5	7xb0	Wildtype, K460N, N405D, N417K, N417T, N477S, Q493K, Q493R, S408R, S486F, S486P, S486V	D420A, D420I, D420L, D420N D420R, F456D, F456L, F456N, F456R, F456V	12	10	22
SFE	XBB.1.5	CryoEM	Wildtype, K460N, N405D, N417K, N417T, N477S, Q493K, Q493R, S408R, S486F, S486P, S486V	D420A, D420I, D420L, D420N D420R, F456D, F456L, F456N,	12	10	22

				F456R, F456V			
Rosetta	BA.2	7xb0	Wildtype, F486P, F486S, F486V, N405D, N417K, N417T, N460K, N477S, R493K, R493Q, S408R	D420A, D420I, D420L, D420N D420R, F456D, F456L, F456N, F456R, F456V	12	10	22
Rosetta	BA.2	CryoEM	Wildtype, F486P, F486S, F486V, N405D, N417K, N417T, N460K, N477S, R493K, R493Q, S408R	D420A, D420I, D420L, D420N D420R, F456D, F456L, F456N, F456R, F456V	12	10	22
Rosetta	XBB	7xb0	Wildtype, K460N, N405D, N417K, N417T, N477S, Q493K, Q493R, S408R, S486F, S486P, S486V	D420A, D420I, D420L, D420N D420R, F456D, F456L, F456N, F456R, F456V	12	10	22
Rosetta	XBB	CryoEM	Wildtype, K460N, N405D, N417K, N417T, N477S, Q493K, Q493R, S408R, S486F, S486P, S486V	D420A, D420I, D420L, D420N D420R, F456D, F456L, F456N, F456R, F456V	12	10	22

FEP Stability	BA.2	N/A	Wildtype	N/A	1	0	1
FEP	BA.2	N/A	Wildtype	D420L,<br>D420N,<br>D420R<br>D420Y<br>F456D,<br>F456L,<br>F456N,<br>F456V	1	8	9
PMF	N/A	N/A	N/A	D420K,<br>F456D	0	2	2
				Total	98	90	188

Table S7. Utility functions used to select 187 sequences in Section S2.2.5.2. Max, Min, and Top N utility functions are described in Table S5.

Utility Function	Repeat	Utility Function	Description of how Utility Function scores a candidate antibody sequence
x1	2	Max (SFE Wildtype)	Worst case binding across all antigen targets, all structural models
2	1	Min (SFE BA.2 Wildtype) s.t Max (SFE BA.2 BT) < 0.2	Best case binding across all structural models for BA.2 such that ddG < 0.2 for all BT targets
3	1	Min (SFE XBB.1.5 Wildtype) s.t Max (SFE XBB.1.5 BT) < 0.2	Best case binding across all structural models for XBB.1.5 such that ddG < 0.2 for all BT targets
4	1	Max Top3 (SFE D420) s.t Max (SFE BT) < 0.2 & Max (SFE Wildtype) < 0.2	Max of the top 3 SFE for D420 antigen variants such that ddG < 0.2 for all BT targets
5	1	Max Top5 (SFE D420) s.t Max (SFE BT) < 0.2 & Max (SFE Wildtype) < 0.2	Max of the top 5 SFE for D420 antigen variants such that ddG < 0.2 for all BT targets
6	1	MaxTop3 (SFE F456) s.t Max (SFE BT) < 0.2 & Max (SFE Wildtype) < 0.2	Max of the top 3 SFE for F456 antigen variants such that ddG < 0.2 for all BT targets
7	1	Max Top5 (SFE F456) s.t Max (SFE BT) < 0.2 & Max (SFE Wildtype) < 0.2	Max of the top 5 SFE for F456 antigen variants such that ddG < 0.2 for all BT targets
8	1	Max Top1 (SFE D420 & F456)	Max of the top 1 SFE for D420 and F456 antigen variants
9	1	Max Top3 (SFE D420 & F456)	Max of the top 3 SFE for D420 and F456 antigen variants
10	1	Max Top5 (SFE D420 & F456)	Max of the top 5 SFE for D420 and F456 antigen variants
11	1	Min (FEP BA.2) s.t Num Mutations = 1 and FEP Stability < 1.0	Best FEP for BA.2 such that FEP Stability less than 1.0, and single point mutation
12	1	Min (FEP BA.2 Conservative) s.t Num Mutations = 1 and FEP Stability < 3.0	Best case binding FEP for BA.2 such that FEP Stability less than 3.0, and single point mutation
13	1	Min (FEP BA.2 Wildtype) s.t FEP Stability < 1.0	Best case binding FEP for BA.2 such that FEP Stability less than 1.0
14	1	Min (FEP BA.2 Wildtype) s.t FEP Stability < 3.0	Best case binding FEP for BA.2 such that FEP Stability less than 3.0
15	1	Max Top2 (FEP D420 & FEP BA.2 Wildtype) s.t FEP Stability < 5.0	Max of the top 2 FEP for D420 antigen variants, BT targets, and BA.2 wildtype such that FEP Stability less than 5.0
16	1	Max Top4 (FEP D420 & FEP BA.2 Wildtype) s.t FEP Stability < 5.0	Top 4 FEP for D420 antigen variants, BT targets and BA.2 wildtype such that FEP Stability less than 5.0
17	1	Max Top2 (FEP F456) s.t FEP Stability < 5.0	Max of the top 2 FEP for F456 antigen variants such that FEP Stability less than 5.0
18	1	Max Top4 (FEP F456) s.t FEP Stability < 5.0	Max of the top 4 FEP for F456 antigen variants such that FEP Stability less than 5.0
19	1	Min (PMF D420)	Best case binding for PMF ddG for D420 antigen mutants
20	1	Min (PMF F456)	Best case binding for PMF ddG for F456 antigen mutants
21	1	Min (Rosetta) s.t Num Mutations = 2	Best 2-point mutation for Rosetta across all antigen targets
22	2	Max (FEP & SFE BA.2) s.t D420 & F456 do not interact	Worst case binding between FEP and SFE in BA.2 background for antibody positions not interacting with D420 and F456.
23	2	Max Top2 (SFE D420 Z-scores & FEP D420 Z-scores & SFE BA.2 Wildtype Z-scores & FEP BA.2 Wildtype Z-scores)	Max of the top 2 Z-scores using SFE and FEP for D420 antigen mutants, BT targets, and BA.2 wildtype
24	2	Max Top4 (SFE D420 Z-scores & FEP D420 Z-scores & SFE BA.2 Wildtype Z-scores & FEP BA.2 Wildtype Z-scores)	Max of the top 4 Z-scores using SFE and FEP for D420 antigen mutants, BT targets, and BA.2 wildtype
25	2	MaxTop2 (SFE F456 Z-scores & FEP F456 Z-scores & SFE BA.2 Wildtype Z-scores & FEP BA.2 Wildtype Z-scores)	Max of the top 2 Z-scores using SFE and FEP for F456 antigen mutants, BT targets, and BA.2 wildtype
26	2	Max Top4 (SFE F456 Z-scores & FEP F456 Z-scores & SFE BA.2 Wildtype Z-scores & FEP BA.2 Wildtype Z-scores)	Max of the top 4 Z-scores using SFE and FEP for F456 antigen mutants, BT targets , and BA.2 wildtype

Table S8. List of combination strategies applied to the sets of mutations identified in the "Select" phase. "Clashes" are defined by the case where two or more antibody mutations are in close proximity with the same AA on the antigen.

Strategy	Number of unique mutants produced
1. Exhaustive combination of T1 mutations up to 3 mutations (n-choose-k with k=1,2,3)	11
2. Exhaustive combination of 2 T1 mutations + 1 ET2 mutations	36
3. Exhaustive combination of 2 T1 mutations + 2 ET2 mutations (clashes included)	24
4. Exhaustive combination of 1 T1 mutation + 2 ET2 mutations (clashes excluded)	26
5. Exhaustive combination of 1 T1 mutation + 3 ET2 mutations (clashes excluded)	6
6. Exhaustive combination of three ET2 mutants	12
7. Exhaustive combination of 1 XS mutation + set generated by "Strategy 1"	6

Table S9. Binding kinetics for AZD3152-parental IgG and AZD3152-1142 IgG to SARS-COV-2 RBDs. K_D was measured via Biolayer Interferometry (BLI) by loading IgG onto anti-human Fc (AHC) sensors followed by association with SARS-COV-2 RBD at varying concentrations. "ND": Kinetic parameters were not determined for measurements where the RBD-antibody association did not reach 0.2 nm at any concentration.

Antibody	RBD	KD (M)	Ka (1/Ms)	Kdis (1/s)	X^2	R^2
AZD3152	XBB.1.5	2.01E-09	4.69E+05	9.44E-04	4.03E+00	9.94E-01
3152-1142	XBB.1.5	9.68E-11	9.85E+05	9.53E-05	1.33E+00	9.91E-01
AZD3152	XBB.1.5 + F456L	ND	ND	ND	ND	ND
3152-1142	XBB.1.5 + F456L	1.72E-08	3.74E+05	6.45E-03	4.30E-01	9.92E-01
AZD3152	KP.3	ND	ND	ND	ND	ND
3152-1142	KP.3	ND	ND	ND	ND	ND

Table S10. The effect of F456L on the potency of AZD3152 is variant-dependent. AZD3152 neutralization potency was measured against pseudoviruses bearing D614G, BA.2, BA.5 or XBB.1.5 spike proteins, with or without a Leu substitution at position 456 (F456L). Fold-change indicates the ratio of the EC_{50} of the variant with F456L, compared to that of the wildtype variant.

Variant		EC_{50} (ng/mL)	Fold-change
D614G	-	6.85	0.9
	+ F456L	5.90	
BA.2	-	6.10	548.4
	+ F456L	3345.25	
BA.5	-	2.17	221.8
	+ F456L	481.21	
XBB.1.5	-	1.92	>7031.3
	+ F456L	>13500	

Data file S1

Design of tiled yeast deep mutagenesis libraries based on XBB.1.5 RBD.

Data file S2

DMS results for XBB.1.5 RBD mutations, including escape scores for ACE2, AZD3152, and 3152-1142.

REFERENCES AND NOTES

1. O. J. Watson, G. Barnsley, J. Toor, A. B. Hogan, P. Winskill, A. C. Ghani, Global impact of the first year of COVID-19 vaccination: A mathematical modelling study. *Lancet Infect. Dis.* **22**, 1293–1302 (2022).

2. Coronavirus pandemic (COVID-19) data explorer. Our World in Data. (December 17, 2023); https://ourworldindata.org/explorers/coronavirus-data-explorer.

3. Y. Wang, B. Cao, The insights from SARS-CoV-2 antibody treatment for future emerging infectious diseases. *Lancet Infect. Dis.* **24**, 2–3 (2024).

4. P. V. Markov, M. Ghafari, M. Beer, K. Lythgoe, P. Simmonds, N. I. Stilianakis, A. Katzourakis, The evolution of SARS-CoV-2. *Nat. Rev. Microbiol.* **21**, 361–379 (2023).

5. Y. Cao, J. Wang, F. Jian, T. Xiao, W. Song, A. Yisimayi, W. Huang, Q. Li, P. Wang, R. An, J. Wang, Y. Wang, X. Niu, S. Yang, H. Liang, H. Sun, T. Li, Y. Yu, Q. Cui, S. Liu, X. Yang, S. Du, Z. Zhang, X. Hao, F. Shao, R. Jin, X. Wang, J. Xiao, Y. Wang, X. S. Xie, Omicron escapes the majority of existing SARS-CoV-2 neutralizing antibodies. *Nature* **602**, 657–663 (2022).

6. D. Planas, N. Saunders, P. Maes, F. Guivel-Benhassine, C. Planchais, J. Buchrieser, W. H. Bolland, F. Porrot, I. Staropoli, F. Lemoine, H. Pere, D. Veyer, J. Puech, J. Rodary, G. Baele, S. Dellicour, J. Raymenants, S. Gorissen, C. Geenen, B. Vanmechelen, T. Wawina-Bokalanga, J. Marti-Carreras, L. Cuypers, A. Seve, L. Hocqueloux, T. Prazuck, F. A. Rey, E. Simon-Loriere, T. Bruel, H. Mouquet, E. Andre, O. Schwartz, Considerable escape of SARS-CoV-2 Omicron to antibody neutralization. *Nature* **602**, 671–675 (2022).

7. Fact Sheet for Patients, Parents And Caregivers Emergency Use Authorization (EUA) of EVUSHELD™ (tixagevimab co-packaged with cilgavimab) for Coronavirus Disease 2019 (COVID-19); https://fda.gov/media/154702/download.

8. FDA. FDA announces Evusheld is not currently authorized for emergency use in the U.S. https://fda.gov/drugs/drug-safety-and-availability/fda-announces-evusheld-not-currently-authorized-emergency-use-us.

9. J. W. Yewdell, Antigenic drift: Understanding COVID-19. *Immunity* **54**, 2681–2687 (2021).

10. B. Fritzell, Bridging studies. *Dev. Biol. Stand.* **95**, 181–188 (1998).

11. T. A. Desautels, K. T. Arrildt, A. T. Zemla, E. Y. Lau, F. Zhu, D. Ricci, S. Cronin, S. J. Zost, E. Binshtein, S. M. Scheaffer, B. Dadonaite, B. K. Petersen, T. B. Engdahl, E. Chen, L. S. Handal, L. Hall, J. W. Goforth, D. Vashchenko, S. Nguyen, D. R. Weilhammer, J. K. Lo, B. Rubinfeld, E. A. Saada, T. Weisenberger, T. H. Lee, B. Whitener, J. B. Case, A. Ladd, M. S. Silva, R. M. Haluska, E. A. Grzesiak, C. G. Earnhart, S. Hopkins, T. W. Bates, L. B. Thackray, B. W. Segelke, C.-C. Tri-lab, A. M. Lillo, S. Sundaram, J. D. Bloom, M. S. Diamond, J. E. Crowe Jr., R. H. Carnahan, D. M. Faissol, Computationally restoring the potency of a clinical antibody against Omicron. *Nature* **629**, 878–885 (2024).

12. T. N. Starr, N. Czudnochowski, Z. Liu, F. Zatta, Y. J. Park, A. Addetia, D. Pinto, M. Beltramello, P. Hernandez, A. J. Greaney, R. Marzi, W. G. Glass, I. Zhang, A. S. Dingens, J. E. Bowen, M. A. Tortorici, A. C. Walls, J. A. Wojcechowskyj, A. De Marco, L. E. Rosen, J. Zhou, M. Montiel-Ruiz, H. Kaiser, J. R. Dillen, H. Tucker, J. Bassi, C. Silacci-Fregni, M. P. Housley, J. di Iulio, G. Lombardo, M. Agostini, N. Sprugasci, K. Culap, S. Jaconi, M. Meury, E. Dellota Jr., R. Abdelnabi, S. C. Foo, E. Cameroni, S. Stumpf, T. I. Croll, J. C. Nix, C. Havenar-Daughton, L. Piccoli, F. Benigni, J. Neyts, A. Telenti, F. A. Lempp, M. S. Pizzuto, J. D. Chodera, C. M. Hebner, H. W. Virgin, S. P. J. Whelan, D. Veesler, D. Corti, J. D. Bloom, G. Snell, SARS-CoV-2 RBD antibodies that maximize breadth and resistance to escape. *Nature* **597**, 97–102 (2021).

13. A. J. Greaney, T. N. Starr, C. O. Barnes, Y. Weisblum, F. Schmidt, M. Caskey, C. Gaebler, A. Cho, M. Agudelo, S. Finkin, Z. Wang, D. Poston, F. Muecksch, T. Hatziioannou, P. D. Bieniasz, D. F. Robbiani, M. C. Nussenzweig, P. J. Bjorkman, J. D. Bloom, Mapping mutations to the SARS-CoV-2 RBD that escape binding by different classes of antibodies. *Nat. Commun.* **12**, 4196 (2021).

14. L. Li, X. Chen, Z. Wang, Y. Li, C. Wang, L. Jiang, T. Zuo, Breakthrough infection elicits hypermutated IGHV3-53/3–66 public antibodies with broad and potent neutralizing activity against SARS-CoV-2 variants including the emerging EG.5 lineages. *PLOS Pathog.* **19**, e1011856 (2023).

15. Y. Cai, S. Diallo, K. Rosenthal, K. Ren, D. J. Flores, A. Dippel, V. Oganesyan, N. van Dyk,X. Chen, E. Cantu, R. Choudhary, M. Sulikowski, H. Adissu, B. Chawla, S. Kar, C. Liu,A. Dijokaite-Guraliuc, J. Mongkolsapaya, S. Rajan, Y. M. Loo, R. Beavon, C. Webber,L. J. Chang, S. Thomas, L. Clegg, H. Zhang, G. R. Screaton, N. Philbin, M. Harre, A. Selim,N. Martinez-Alier, A. Uriel, T. S. Cohen, J. L. Perez, M. T. Esser, W. Blair, J. R. Francica, AZD3152 neutralizes SARS-CoV-2 historical and contemporary variants and is protective in hamsters and well tolerated in adults. *Sci. Transl. Med.* **16**, eado2817 (2024).

16. F. Zhu, F. A. Bourguet, W. F. D. Bennett, E. Y. Lau, K. T. Arrildt, B. W. Segelke, A. T. Zemla, T. A. Desautels, D. M. Faissol, Large-scale application of free energy perturbation calculations for antibody design. *Sci. Rep.* **12**, 12489 (2022).

17. S. Conti, E. Y. Lau, V. Ovchinnikov, On the rapid calculation of binding affinities for antigen and antibody design and affinity maturation simulations. *Antibodies* **11**, 51 (2022).

18. K. A. Barlow, S. Ó. Conchúir, S. Thompson, P. Suresh, J. E. Lucas, M. Heinonen, T. Kortemme, Flex ddG: Rosetta ensemble-based estimation of changes in protein-protein binding affinity upon mutation. *J. Phys. Chem. B* **122**, 5389–5399 (2018).

19. A. Zemla, T. Desautels, E. Y. Lau, F. Zhu, K. T. Arrildt, B. W. Segelke, S. Sundaram, D. Faissol, SARS-COV-2 Omicron variant predicted to exhibit higher affinity to ACE-2 receptor and lower affinity to a large range of neutralizing antibodies, using a rapid computational platform. bioRxiv. 472843 [Preprint] (2021). https://doi.org/10.1101/2021.12.16.472843.

20. F. Jian, L. Feng, S. Yang, Y. Yu, L. Wang, W. Song, A. Yisimayi, X. Chen, Y. Xu, P. Wang, L. Yu, J. Wang, L. Liu, X. Niu, J. Wang, T. Xiao, R. An, Y. Wang, Q. Gu, F. Shao, R. Jin, Z. Shen, Y. Wang, X. Wang, Y. Cao, Convergent evolution of SARS-CoV-2 XBB lineages on receptor-binding domain 455-456 synergistically enhances antibody evasion and ACE2 binding. *PLOS Pathog.* **19**, e1011868 (2023).

21. E. Baldwin, P. G. Schultz, Generation of a catalytic antibody by site-directed mutagenesis. *Science* **245**, 1104–1107 (1989).

22. F. Zhao, M. Yuan, C. Keating, N. Shaabani, O. Limbo, C. Joyce, J. Woehl, S. Barman, A. Burns, Q. Tran, X. Zhu, M. Ricciardi, L. Peng, J. Smith, D. Huang, B. Briney, D. Sok, D. Nemazee, J. R. Teijaro, I. A. Wilson, D. R. Burton, J. G. Jardine, Broadening a SARS-CoV-1-neutralizing antibody for potent SARS-CoV-2 neutralization through directed evolution. *Sci. Signal.* **16**, eabk3516 (2023).

23. L. Witte, V. A. Baharani, F. Schmidt, Z. Wang, A. Cho, R. Raspe, C. Guzman-Cardozo,F. Muecksch, M. Canis, D. J. Park, C. Gaebler, M. Caskey, M. C. Nussenzweig,T. Hatziioannou, P. D. Bieniasz, Epistasis lowers the genetic barrier to SARS-CoV-2 neutralizing antibody escape. *Nat. Commun.* **14**, 302 (2023).

24. A. L. Taylor, T. N. Starr, Deep mutational scans of XBB.1.5 and BQ.1.1 reveal ongoing epistatic drift during SARS-CoV-2 evolution. *PLOS Pathog.* **19**, e1011901 (2023).

25. A. M. Carabelli, T. P. Peacock, L. G. Thorne, W. T. Harvey, J. Hughes, C.-G. U. Consortium, S. J. Peacock, W. S. Barclay, T. I. de Silva, G. J. Towers, D. L. Robertson, SARS-CoV-2 variant biology: Immune escape, transmission and fitness. *Nat. Rev. Microbiol.* **21**, 162–177 (2023).

26. C. Liu, H. M. Ginn, W. Dejnirattisai, P. Supasa, B. Wang, A. Tuekprakhon, R. Nutalai, D. Zhou, A. J. Mentzer, Y. Zhao, H. M. E. Duyvesteyn, C. Lopez-Camacho, J. Slon-Campos, T. S. Walter, D. Skelly, S. A. Johnson, T. G. Ritter, C. Mason, S. A. C. Clemens, F. G. Naveca, V. Nascimento, F. Nascimento, C. F. da Costa, P. C. Resende, A. Pauvolid-Correa, M. M. Siqueira, C. Dold, N. Temperton, T. Dong, A. J. Pollard, J. C. Knight, D. Crook, T. Lambe, E. Clutterbuck, S. Bibi, A. Flaxman, M. Bittaye, S. Belij-Rammerstorfer, S. C. Gilbert, T. Malik, M. W. Carroll, P. Klenerman, E. Barnes, S. J. Dunachie, V. Baillie, N. Serafin, Z. Ditse, K. Da Silva, N. G. Paterson, M. A. Williams, D. R. Hall, S. Madhi, M. C. Nunes, P. Goulder, E. E. Fry, J. Mongkolsapaya, J. Ren, D. I. Stuart, G. R. Screaton, Reduced neutralization of SARS-CoV-2 B.1.617 by vaccine and convalescent serum. *Cell* **184**, 4220–4236.e13 (2021).

27. S. B. Geng, J. Wu, M. E. Alam, J. S. Schultz, C. D. Dickinson, C. R. Seminer, P. M. Tessier, Facile preparation of stable antibody-gold conjugates and application to affinity-capture self-interaction nanoparticle spectroscopy. *Bioconjug. Chem.* **27**, 2287–2300 (2016).

28. L. Li, H. Liao, Y. Meng, W. Li, P. Han, K. Liu, Q. Wang, D. Li, Y. Zhang, L. Wang, Z. Fan, Y. Zhang, Q. Wang, X. Zhao, Y. Sun, N. Huang, J. Qi, G. F. Gao, Structural basis of human ACE2 higher binding affinity to currently circulating Omicron SARS-CoV-2 sub-variants BA.2 and BA.1.1. *Cell* **185**, 2952–2960.e10 (2022).

29. W. L. Jorgensen, J. Chandrasekhar, J. D. Madura, R. W. Impey, M. L. Klein, Comparison of simple potential functions for simulating liquid water. *J. Chem. Phys.* **79**, 926–935 (1983).

30. V. Ovchinnikov, S. Conti, E. Y. Lau, F. C. Lightstone, M. Karplus, Microsecond molecular dynamics simulations of proteins using a quasi-equilibrium solvation shell model. *J. Chem. Theory Comput.* **16**, 1866–1881 (2020).

31. D. A. Case, I. Y. Ben-Shalom, S. R. Brozell, D. S. Cerutti, T. E. Cheatham III, V. W. D. Cruzeiro, T. A. Darden, R. E. Duke, D. Ghoreishi, M. K. Gilson, H. Gohlke, A. W. Goetz, D. Greene, R. Harris, N. Homeyer, Y. Huang, S. Izadi, A. Kovalenko, T. Kurtzman, T. S. Lee, S. LeGrand, P. Li, C. Lin, J. Liu, T. Luchko, R. Luo, D. J. Mermelstein, K. M. Merz, Y. Miao, G. Monard, C. Nguyen, H. Nguyen, I. Omelyan, A. Onufriev, F. Pan, R. Qi, D. R. Roe, A. Roitberg, C. Sagui, S. Schott-Verdugo, J. Shen, C. L. Simmerling, J. Smith, R. Salomon-Ferrer, J. Swails, R. C. Walker, J. Wang, H. Wei, R. M. Wolf, X. Wu, L. Xiao, D. M. York, P. A. Kollman. AMBER, version 18 (University of California, San Francisco, CA, 2018).

32. J. A. Maier, C. Martinez, K. Kasavajhala, L. Wickstrom, K. E. Hauser, C. Simmerling, ff14SB: Improving the accuracy of protein side chain and backbone parameters from ff99SB. *J. Chem. Theory Comput.* **11**, 3696–3713 (2015).

33. S. Jemimah, M. M. Gromiha, Exploring additivity effects of double mutations on the binding affinity of protein-protein complexes. *Proteins* **86**, 536–547 (2018).

34. F. L. Silva, J. Yang, M. Landajuela, A. Goncalves, A. Ladd, D. Faissol, B. Petersen, Toward multi-fidelity reinforcement learning for symbolic optimization, paper presented at the Adaptive and Learning Agents (ALA) Workshop @ AAMAS, London, England, 2023.

35. J. Faris, C. Hayes, A. Goncalves, K. Sprenger, D. Faissol, M. Landajuela, B. Petersen, F. L. Silva, Pareto front training for multi-objective symbolic optimization, paper presented at the Adaptive and Learning Agents (ALA) Workshop @ AAMAS, Auckland, New Zealand, 2024.

36. D. M. Roijers, P. Vamplew, S. Whiteson, R. Dazeley, A survey of multi-objective sequential decision-making. *J Artif Intell Res* **48**, 67–113 (2013).

37. C. F. Hayes, R. Radulescu, E. Bargiacchi, J. Källström, M. Macfarlane, M. Reymond, T. Verstraeten, L. M. Zintgraf, R. Dazeley, F. Heintz, E. Howley, A. A. Irissappane, P. Mannion, A. Nowé, G. Ramos, M. Restelli, P. Vamplew, D. M. Roijers, A practical guide to multi-objective reinforcement learning and planning. *Auton. Agent. Multi-Agent Syst.* **36**, 26 (2022).

38. R. Tibshirani, Regression shrinkage and selection via the Lasso. *J. R. Stat. Soc. Series B Stat. Methodol.* **58**, 267–288 (1996).
